# Heterostructure-Engineered Semiconductor Quantum Dots toward Photocatalyzed-Redox Cooperative Coupling Reaction

**DOI:** 10.34133/research.0073

**Published:** 2023-03-10

**Authors:** Lin-Xing Zhang, Ming-Yu Qi, Zi-Rong Tang, Yi-Jun Xu

**Affiliations:** College of Chemistry, State Key Laboratory of Photocatalysis on Energy and Environment, Fuzhou University, Fuzhou 350116, P.R. China.

## Abstract

Semiconductor quantum dots have been emerging as one of the most ideal materials for artificial photosynthesis. Here, we report the assembled ZnS-CdS hybrid heterostructure for efficient coupling cooperative redox catalysis toward the oxidation of 1-phenylethanol to acetophenone/2,3-diphenyl-2,3-butanediol (pinacol) integrated with the reduction of protons to H_2_. The strong interaction and typical type-I band-position alignment between CdS quantum dots and ZnS quantum dots result in efficient separation and transfer of electron-hole pairs, thus distinctly enhancing the coupled photocatalyzed-redox activity and stability. The optimal ZnS-CdS hybrid also delivers a superior performance for various aromatic alcohol coupling photoredox reaction, and the ratio of electrons and holes consumed in such redox reaction is close to 1.0, indicating a high atom economy of cooperative coupling catalysis. In addition, by recycling the scattered light in the near field of a SiO_2_ sphere, the SiO_2_-supported ZnS-CdS (denoted as ZnS-CdS/SiO_2_) catalyst can further achieve a 3.5-fold higher yield than ZnS-CdS hybrid. Mechanistic research clarifies that the oxidation of 1-phenylethanol proceeds through the pivotal radical intermediates of ^•^C(CH_3_)(OH)Ph. This work is expected to promote the rational design of semiconductor quantum dots-based heterostructured catalysts for coupling photoredox catalysis in organic synthesis and clean fuels production.

## Introduction

Over the past decades, semiconductor quantum dots (QDs), featuring unique electrical and optical properties related to size and shape, have been identified as one of the most promising materials for various applications in biomedical, optoelectronic devices and photocatalysis [1–5]. QDs with characteristic sizes smaller than the exciton Bohr radius, and the electrons and holes are confined by potential barriers in 3 spatial dimensions, which makes them quite different from traditional bulk photocatalytic materials [6,7]. For example, the quantum confinement effect endows a semiconductor with broadened bandgap, which supplies a more marked driving force for redox reactions [8–10]. Also, the processes, termed as multiple-exciton generation, lay the foundation for driving multiple-electrons redox reactions over QDs [11,12]. The enormous surface-to-volume ratio and insufficient surface atom coordination allow for the formation of heterostructured hybrid, thereby shortening their charge transfer distance [13,14].

As a typical representative of metal chalcogenide QDs, CdS QDs possess suitable bandgap positions to meet the requirements of redox potential, i.e., the conduction band (CB) position is sufficiently negative (~ −0.9 V vs. normal hydrogen electrode [NHE]) for hydrogen (H_2_) evolution, and the valence band (VB) position has a positive enough potential to oxidize typical organic substrates [15,16]. Recent years have witnessed the win-win coupling strategy of photocatalyzed-redox reaction in one system that utilizes electrons and holes simultaneously for integrating selective organic synthesis and H_2_ evolution [17,18]. To conquer the severe charge recombination and lack of surface-active sites in CdS QDs-based photocatalysts, several attempts have been explored to improve the catalytic performance [13,19,20]. For example, Lian and coworkers synthesized CdSe/CdS heterostructured core/shell QDs capable of transferring multiple electrons to surface-absorbed methylviologen molecules, confirming that intimate heterointerface contact over heterostructured QDs maintains an ultrafast charge separation rate and slow recombination [21].

Herein, we for the first time have reported heterostructured ZnS-CdS hybrid for efficient oxidation of 1-phenylethanol to acetophenone and 2,3-diphenyl-2,3-butanediol (pinacol) integrated with H_2_ evolution. The close interfacial contact with a short charge transport distance is obtained in the assembled aggregates, which enables a valid type-I interfacial charge transfer between CdS QDs and ZnS QDs. Consequently, the ZnS-CdS hybrid exhibits an increased photocatalyzed-redox activity and stability for the coproduction of acetophenone/pinacol and H_2_. Besides, in comparison with ZnS-CdS hybrid, the SiO_2_-supported ZnS-CdS (ZnS-CdS/SiO_2_) can further realize a 3.5-fold activity enhancement by employing the scattered light in the near field of a SiO_2_ sphere. Mechanistic studies based on control experiments and electron paramagnetic resonance (EPR) spectroscopy reveal the carbon-centered radicals as the key active intermediates for the photocatalytic reaction. We hope that the present work will provide insights into the construction of QDs-based heterostructured photocatalysts with suitable redox potentials for efficient coupling of photocatalyzed-redox reactions.

## Results and Discussion

**Fig. 1.**
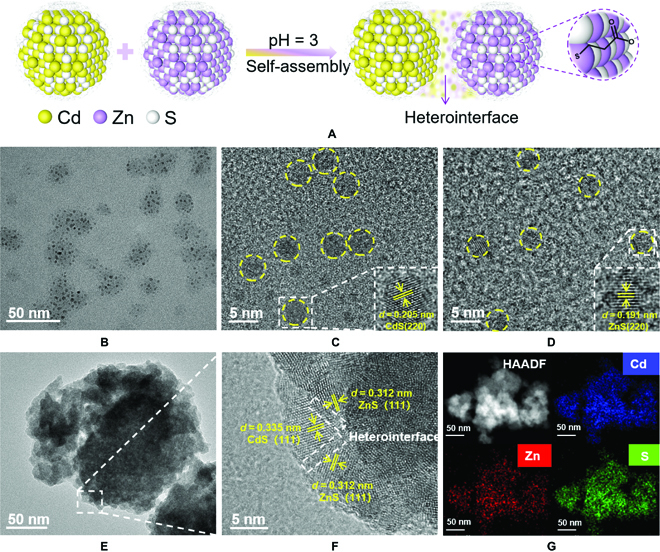
Synthetic process diagram and morphological characterization. (A) Schematic diagram of ZnS-CdS hybrid prepared via self-assembly process. (B) TEM image of CdS QDs. HRTEM images of (C) CdS QDs and (D) ZnS QDs. (E) TEM image, (F) HRTEM image, and (G) high-angle annular dark field (HAADF) and corresponding elemental mapping results of ZnS-CdS hybrid.

The fabrication route of ZnS-CdS hybrids by self-assembly procedure is portrayed in Fig. 1A. To begin with, CdS QDs are prepared by reacting CdCl_2_ with Na_2_S in aqueous solution with the existence of 3-mercaptopropionic acid (MPA) as capping ligands. The preparation steps of ZnS QDs take the same method, except for the difference of precursor salts (ZnCl_2_ for ZnS QDs) (Fig. S1). According to the transmission electron microscopy (TEM) (Fig. 1B) and high-resolution TEM (HRTEM) (Fig. 1C) observation, the as-prepared CdS QDs have an average diameter of about 5 nm (Fig. S2) and a lattice distance of 0.205 nm, which attributes to the (220) facet. The ZnS QDs exhibit smaller size distribution (~4 nm) with a lattice fringe distance of 0.191 nm assigned to the (220) facet of cubic phase ZnS (Fig. 1D and Fig. S3A). Furthermore, to construct heterostructure of ZnS-CdS hybrids, the pH of the solution from alkaline to acidic conditions (pH = 3.0) is regulated by adding HCl (Fig. 1A). In this process, the separation of partial ligands from the QDs surface results in the loss of electrostatic repulsion between the QDs and promotes spontaneous assembly into inorganic aggregates [22,23].

As shown in Fig. 1E and F and Fig. S3B, the ZnS-CdS hybrid gives a larger size distribution with many overlapping areas, suggesting the formation of assembled aggregates. The phase margin forms a region of crystalline disorder with intense lattice distortion, which means that there is a fierce interfacial interaction between CdS and ZnS QDs in the assembled aggregates [24]. Besides, the lattice fringes of 0.335 and 0.312 nm referring to the (111) facets of CdS and ZnS can be clearly observed, which may be attributed to the acidity-induced crystal facet exposure [25–27]. The corresponding selected-area electron diffraction (Fig. S3C) shows that the index of the primary rings are consistent with the presence of (111) facets of CdS and ZnS, respectively [25]. These results confirm the successful construction of heterostructured ZnS-CdS hybrid with intimate interfacial interactions [28]. Further evidence derives from energy-dispersive X-ray spectroscopy (EDX) and corresponding elemental mapping analysis (Fig. S4 and Fig. 1G). All the characteristic elements including Cd, Zn, S, and O are homogeneously distributed in the assembled aggregates, suggesting that CdS QDs and ZnS QDs are uniformly hybridized together.

**Fig. 2.**
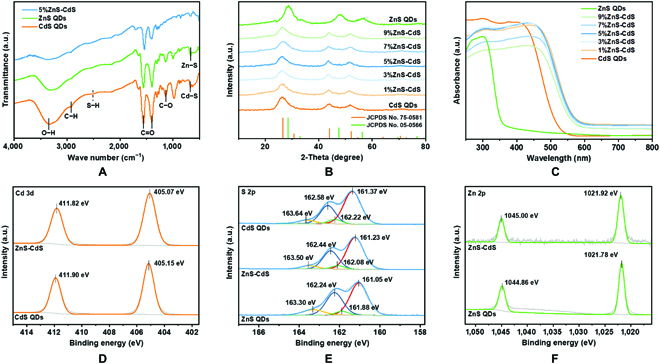
Physicochemical properties. (A) FTIR spectra of MPA-capped CdS QDs, ZnS QDs, and ZnS-CdS hybrid. (B) XRD patterns and (C) DRS spectra of CdS QDs, ZnS QDs, and ZnS-CdS hybrids. XPS spectra for CdS QDs, ZnS QDs, and ZnS-CdS hybrid: (D) Cd 3d, (E) S 2p, and (F) Zn 2p. a.u., arbitrary units; JCPDS, Joint Committee on Powder Diffraction Standards.

The obtained MPA-capped QDs are then analyzed through Fourier transform infrared spectroscopy (FTIR) (Fig. 2A) to verify that partial ligands are removed from the QDs surface after self-assembly. In the hybrid, the decreased intensity of asymmetrical and symmetric stretching vibration of the C=O, and O−H stretching vibration at 1,562, 1,400, and 3,310 cm^−1^, demonstrating that the successful formation of assembled aggregates [16,29]. Moreover, the absence of S−H stretching vibration around 2,500 cm^−1^ illustrates that thiol groups form a strong covalent bound with Cd^2+^ and Zn^2+^ on the surface of QDs [30]. In the X-ray diffraction (XRD) patterns (Fig. 2B), the diffraction peaks of CdS QDs (Joint Committee on Powder Diffraction Standards No. 75-0581) are located at 26.55, 44.05, and 52.17°, which belong to (111), (220), and (311) lattice facets, respectively. No obvious characteristic peaks related to ZnS can be observed in the XRD pattern of ZnS-CdS hybrids, which may be owing to the low weight contents of ZnS in the hybrids. Ultraviolet-visible (UV-vis) diffuse reflectance spectroscopy (DRS) has been measured to study the optical properties of all the as-prepared samples. As displayed in Fig. 2C, the strong absorption edges of bare CdS and ZnS QDs are located near 520 and 360 nm, respectively [31]. As for the ZnS-CdS hybrids, the absorption edges display a slight redshift (~580 nm) compared to that of CdS QDs, which may be due to the strong interfacial interaction between CdS and ZnS QDs in the assembled aggregates [32,33].

X-ray photoelectron spectroscopy (XPS) has been performed to explore the surface chemical state of ZnS-CdS hybrid. As shown in the XPS survey spectra (Fig. S5), all the characteristic elements including Cd, Zn, S and O localize on the hybrid, which is in line with the results of EDX spectrum and element mapping. The decreased peak areas of C–C/C–H, C=O, and C–O associated with –COOH functional group in C 1s of the hybrid are well matched with FTIR results (Table S1). In Fig. 2D, the high-resolution Cd 3d spectra of ZnS-CdS hybrid show 2 peaks at 411.82 and 405.07 eV, corresponding to the binding energies of Cd 3d_3/2_ and Cd 3d_5/2_, respectively. Compared to bare CdS QDs, these peaks are negatively shifted by 0.08 eV. A similar negative shift (0.14 eV) appears in the S 2p spectra, while these peaks are located at higher values than bare ZnS QDs (Fig. 2E). Additionally, the S 2p spectra can be split into 4 peaks, of which the binding energies are 163.50 eV (S 2p_1/2_) and 162.44 eV (S 2p_3/2_) derived from precursor thiolate, and the S^2−^ in CdS and ZnS lattice located at 162.08 eV (S 2p_1/2_) and 161.23 eV (S 2p_3/2_) [15,34]. Correspondingly, the binding energies of Zn 2p_1/2_ (1,045.00 eV) and Zn 2p_3/2_ (1,021.92 eV) are 0.14 eV higher than those of bare ZnS QDs (Fig. 2F), indicating that the electron migrates from ZnS to CdS after hybridization [35,36]. The above results show the formation of strong chemical interaction between ZnS and CdS in the self-assembled heterostructure.

**Fig. 3.**
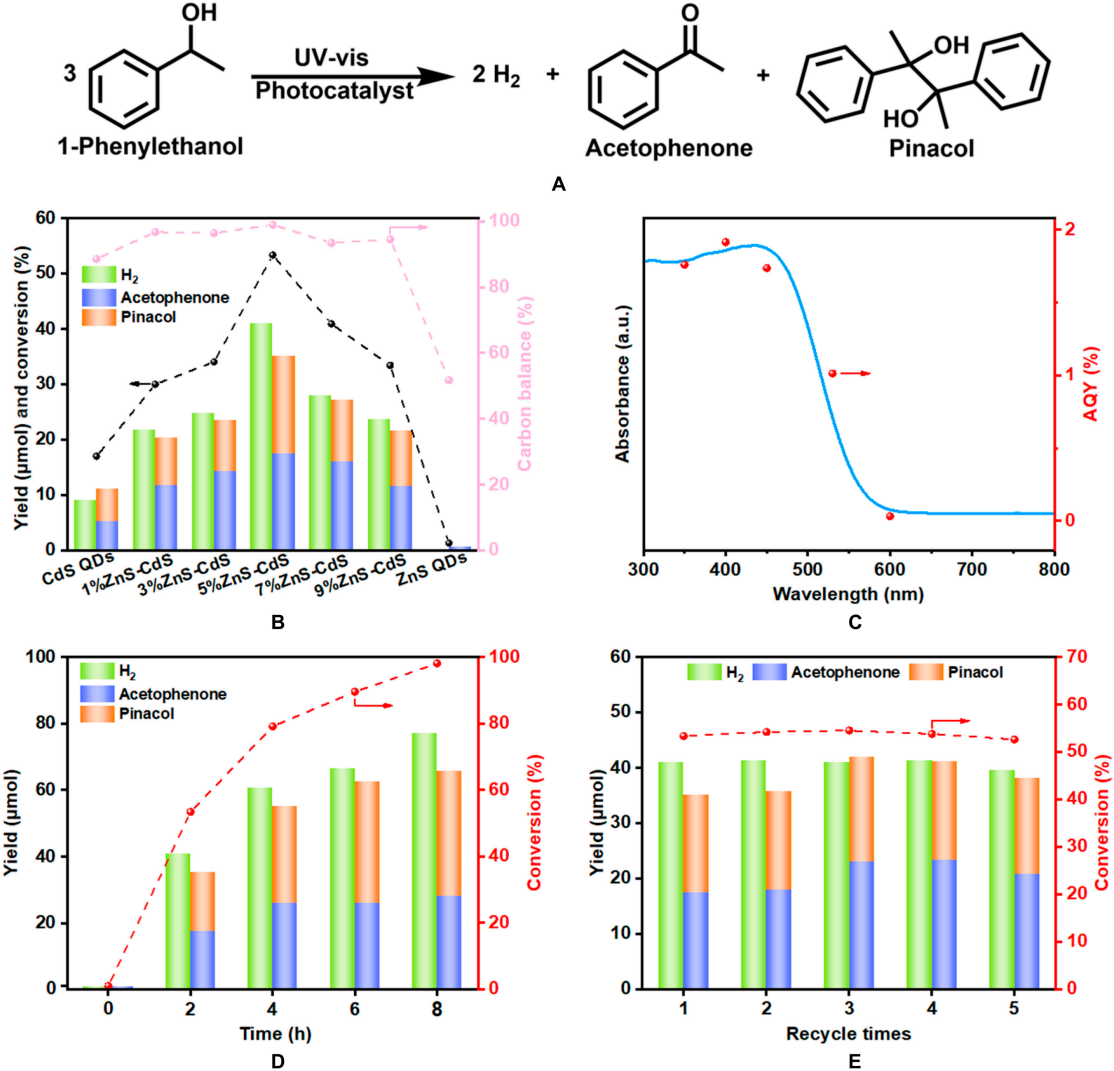
Photocatalyzed-redox 1-phenylethanol paired with H_2_ evolution reaction. (A) The formula for coupling photocatalyzed-redox 1-phenylethanol to acetophenone/pinacol integrated with H_2_ evolution. (B) Photocatalytic activities of CdS QDs, ZnS QDs, and ZnS-CdS hybrids. (C) DRS spectrum of 5%ZnS-CdS hybrid and AQYs of H_2_ under various monochromatic lights. (D) Long-term photocatalytic activity tests and (E) recycling photocatalytic activity tests over 5%ZnS-CdS hybrid.

Considering that acetophenone and 2,3-diphenyl-2,3-butanediol (pinacol) are important structural motifs in fine chemicals and organic chemical intermediates, the selective oxidation of 1-phenylethanol has attracted extensive research interest [37]. The photocatalyzed-redox coproduction of acetophenone/pinacol and H_2_ over the obtained samples has been examined in an Ar-saturated 1-phenylethanol solution with CH_3_CN as solvent (Fig. 3A). The main oxidation products (acetophenone and pinacol) are identified by gas chromatography-mass spectrometry (GC-MS) and quantified by high-performance liquid chromatography (HPLC) methods (Figs. S6 and S7). As shown in Fig. 3B, both bare CdS and ZnS QDs exhibit relatively low photocatalytic activity, mainly due to the rapid recombination of electron-hole pairs, and acetophenone is detected as the main product over ZnS QDs. The photocatalytic activities of the hybrids follow a volcano-type trend, with the maximum performance observed at 5%. Specifically, the 5%ZnS-CdS hybrid with the outputs of all products are 76.08 μmol (40.93 μmol of H_2_, 17.50 μmol of acetophenone, and 17.65 μmol of pinacol) after 2 h of illumination, which is about 3.8 times larger than those of corresponding bare CdS QDs (9.13 μmol of H_2_, 5.29 μmol of acetophenone, and 5.76 μmol of pinacol). Moreover, the yield of oxidation products (acetophenone and pinacol) and the reduction products (H_2_) is close to 1:1, and the carbon balance of this reaction is higher than 93%, indicating a stoichiometric reaction. Notably, the apparent quantum yields (AQYs) over 5%ZnS-CdS hybrid for H_2_ production are tightly connected with the wavelength of incident light, matching well with its DRS spectrum, and the optimized AQY of 1.91% is achieved at λ = 400 nm (Fig. 3C).

When considering the stability and reusability of the catalyst, long-term experiments and recycling tests have been performed on 5%ZnS-CdS hybrid. As displayed in Fig. 3D, the total product yields are 142.60 μmol (76.93 μmol for H_2_, 28.06 μmol for acetophenone, and 37.61 μmol for pinacol), and the conversion of 1-phenylethanol can reach 98% after 8 h of continuous irradiation. Besides, after 5 cycles of tests (Fig. 3E), no distinct changes can be seen on the 5%ZnS-CdS hybrid catalyst. Especially, no marked difference in the crystal structure and light-capturing properties of fresh and used 5%ZnS-CdS hybrid (Fig. S8) can be seen. The results of HRTEM image and element mapping (Fig. S9) also confirm the good stability of 5%ZnS-CdS hybrid during the photocatalytic reaction process. However, the conversion of bare CdS QDs was reduced by 16.36% after 5 cycles of tests (Fig. S10). The above results demonstrate that heterostructured hybrid can significantly ameliorate the photochemical stability of CdS QDs.

Furthermore, to enhance the light-harvesting property of the catalyst, we have assembled the ZnS-CdS hybrid onto the spherical SiO_2_ support to recover the scattered light in the near field of SiO_2_ [16]. As depicted in Fig. S11, the hybrids are evenly distributed on the surface of the SiO_2_ sphere, with an average thickness of ~10 nm. The lattice fringes of 0.335 and 0.312 nm can be attributed to the (111) crystal facet of CdS and ZnS QDs, respectively, which are consistent with the results of 5%ZnS-CdS hybrid (Fig. 1F). DRS spectra (Fig. S12) of 5%ZnS-CdS/SiO_2_ composite show 5 well-defined absorption peaks at ~236, 274, 315, 360, and 410 nm that can be resolved. Nevertheless, neither SiO_2_ nor branched poly-ethylenimine (BPEI)-SiO_2_ has observed these peaks, suggesting that the appearance of these characteristic absorption peaks for 5%ZnS-CdS/SiO_2_ composite can be assigned to the existence of the 5%ZnS-CdS hybrid [38]. As expected, the 5%ZnS-CdS/SiO_2_ composite shows significantly enhanced catalytic performance (Fig. S13) with a total product yield of up to 52.50 mmol g^−1^, which is 3.5- and 13-fold of 5%ZnS-CdS hybrid and bare CdS QDs, respectively. This can be interpreted as the optical absorption model [39,40] promoted by the near-field scattering can flexibly adjust the optical trapping ability of hybrid for coupling photocatalyzed-redox of 1-phenylethanol dehydrogenation.

To verify the generality of ZnS-CdS hybrid material for the oxidation of aromatic alcohols, we have expanded the assay of the alcohol substrates, including benzyl alcohol, 4-methylbenzyl alcohol, 4-methoxybenzyl alcohol, 4-chlorobenzyl alcohol, and 4-nitrobenzyl alcohol as presented in Table 1 (entries 2 to 6 and corresponding characterizations in Figs. S14 to S18). The 5%ZnS-CdS hybrid can effectively oxidize these diverse aromatic alcohols to their corresponding aldehydes/pinacols paired with H_2_ release. Further studies have demonstrated that the introduction of electron-donating groups on the aromatic ring can markedly improve the reaction yield. For example, excellent yields of aldehyde/pinacol are obtained with a methyl group (–CH_3_) or a methoxy group (–OCH_3_) at the para-position. In contrast, the benzyl alcohol with electron-withdrawing groups (–Cl and –NO_2_) greatly decreased the yield (entries 5 and 6 in Table 1) [41].

**Table 1.**
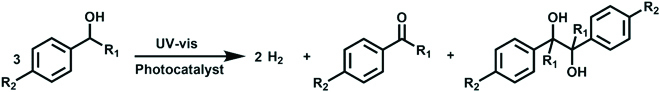
Summary of photocatalytic oxidation of alcohol substrates integrated with H_2_ evolution ^a^.

Entry	–R_1_	–R_2_	H_2_ (μmol)	Liquid products (μmol)		Conversion(%) ^b^	e^−^/h^+^^c^
				Ketone/aldehyde	Pinacol		
1	–CH_3_	–H	76.93	28.06	37.61	98.07	1.17
2	–H	–H	47.28	3.53	47.50	96.68	0.93
3	–H	–CH_3_	53.69	1.03	49.12	99.14	1.07
4	–H	–OCH_3_	90.12	97.72	–	99.70	0.92
5	–H	–Cl	35.57	2.28	37.96	79.76	0.88
6	–H	–NO_2_	17.34	20.74	–	23.59	0.84

^a^ Reaction conditions: Five milligrams of catalyst was dispersed in 5 ml of CH_3_CN, and 0.1 mmol of 1-phenylethanol was added to the solution. Then, Ar was purged into the solution for 20 min to expel the air inside and illuminated with a 300-W Xe arc lamp (300 nm ≤ λ ≤ 800 nm) for 8 h at room temperature. ^b^ Conversion of aromatic alcohol was calculated according to equation of [*n*_0_(alcohol) − *n*(alcohol)]/*n*_0_(alcohol) × 100%. ^c^ The ratio of electrons and holes consumed in redox reactions was calculated by the following equation: e^−^/h^+^ = *n*(H_2_)/[(*n*(acetophenone/aldehyde) + *n*(pinacol)].

**Fig. 4.**
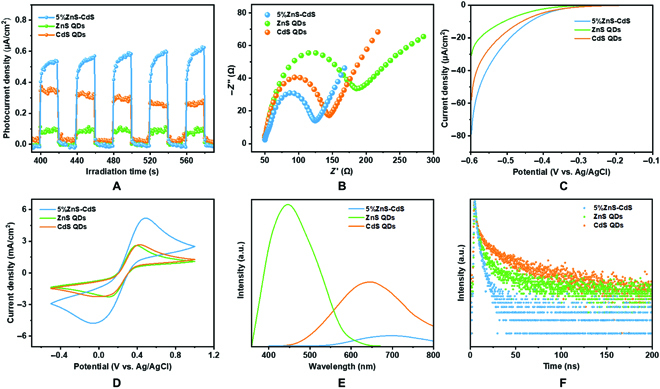
The performance of the charge separation. (A) Transient photocurrent spectra, (B) EIS Nyquist plots, (C) polarization curves, (D) cyclic voltammetry curves, (E) steady-state PL emission spectra, and (F) time-resolved PL decay plots of CdS QDs, ZnS QDs, and 5%ZnS-CdS hybrid.

To disclose the reason for the enhanced photocatalytic performance of ZnS-CdS hybrids in comparison with bare CdS and ZnS QDs, we examined the separation and transfer capabilities of photogenerated electron-hole pairs through photoelectrochemical, electrochemical, and photoluminescence (PL) tests. As shown in Fig. 4A, the transient photocurrent measurement was performed in 0.2 M Na_2_SO_4_ aqueous solution (pH = 6.8) without voltage bias, and the 5%ZnS-CdS hybrid displays the highest photocurrent (0.5 μA/cm^2^), which is about 1.4- and 5-fold higher than that of bare CdS QDs (0.35 μA/cm^2^) and ZnS QDs (0.1 μA/cm^2^), respectively. As expected, the hybrid possesses the smallest electrochemical impedance spectroscopy (EIS) semicircle radius (Fig. 4B), implying a lower resistance and higher charge migration and separation efficiency [42]. The polarization curves of 5%ZnS-CdS hybrid display an enhanced onset overpotential, thus promoting proton reduction to H_2_ (Fig. 4C). As portrayed in the cyclic voltammetry curves (Fig. 4D), the hybrid also shows the enhancement of current density, which is in accordance with the results of EIS Nyquist plots. Furthermore, PL spectra have been performed to analyze the interfacial charge transfer property. In Fig. 4E and Fig. S19, bare CdS QDs exhibit a strong fluorescence peak at 642 nm (380-nm excitation), and a little red shift (~700 nm) in line with the absorption edge is observed in the hybrid (Fig. 2C) [35,43]. Clearly, the PL intensity of 5%ZnS-CdS hybrid quenches significantly, demonstrating that the recombination of electron-hole pairs in CdS is strongly suppressed by the formation of heterostructure [24]. This conclusion is also endorsed by the time-resolved PL decay plots, in which the hybrid also displays accelerated decay behavior (Fig. 4F). Fitting the decay plots by exponential decay kinetics function (Table S2), it is found that the average emission lifetime of 5%ZnS-CdS hybrid is drastically shortened from 2.46 ns of CdS QDs to 1.22 ns, implying the faster interfacial charge transfer occurring in the self-assembled aggregates [44,45]. All the above analysis results indicate the existence of an effective charge transfer in the ZnS-CdS heterostructure, which substantially suppresses electron-hole recombination [33,46].

**Fig. 5.**
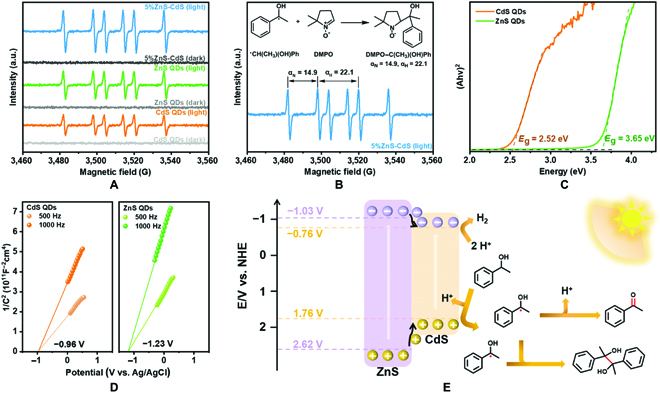
The mechanism of photocatalyzed-redox reaction. (A) EPR spectroscopy in an Ar-saturated 1-phenylethanol solution of CdS QDs, ZnS QDs, and 5%ZnS-CdS hybrid suspensions with or without light illumination (300 nm ≤ λ ≤ 800 nm) and (B) corresponding quantitative analysis results of 5%ZnS-CdS hybrid. (C) Tauc plots and (D) Mott-Schottky plots of CdS QDs and ZnS QDs. (E) Proposed type-I reaction mechanism for coupling photocatalyzed-redox reaction over heterostructured ZnS-CdS hybrid.

To investigate the reaction mechanism of the coupled photocatalyzed-redox 1-phenylethanol reaction, we have carried out controlled experiments, as shown in Table 2. The reaction does not proceed at all in lack of light illumination or catalyst, implying that the reaction is a light-driven process (entries 1 to 3). Besides, adding carbon tetrachloride (CCl_4_) as an electron sacrificial agent to the reaction system significantly inhibits the evolution of H_2_, indicating that the formation of H_2_ is a process of consuming photogenerated electrons (entry 4). Almost no oxidation products (acetophenone and pinacol) are detected when the hole sacrificial agent triethanolamine (TEOA) is introduced into the reaction system (entry 5) due to the key role of holes in 1-phenylethanol oxidation, while a small amount of H_2_ probably originated from TEOA dehydrogenation. Furthermore, using 5,5-dimethyl-1-pyrroline *N*-oxide (DMPO) as free radicals capture agent (entry 6) abruptly reduces photocatalytic activity and the C–C coupling product pinacol cannot be detected, demonstrating that the presence of radical intermediates during the reaction. Thereafter, EPR spectroscopy is used to identify the free radical intermediates of the reaction. Figure 5A and B displays that all 3 samples show 6 peaks with the same relative intensity under simulated sunlight illumination. In nitroxide nitrogen, the nitrogen (α_N_) and hydrogen hyperfine splitting (α_H_) are 14.9 and 22.1, respectively, which are assigned to DMPO–C(CH_3_)(OH)Ph [47,48]. Liquid chromatography-mass spectrometry (Fig. S20) confirms the formation of the DMPO–Cα radical adduct. Furthermore, the higher DMPO–Cα radicals signal intensities in 5%ZnS-CdS hybrid indicate that the hybrid catalytic system is beneficial to generating a large number of such Cα radicals, therefore improving the photocatalytic activity [16].

**Table 2.** Controlled experiment results for coupling photocatalyzed-redox reaction of 1-phenylethanol integrated with H_2_ evolution ^a^.

Entry	Light source	Quenchers	H_2_ (μmol)	Liquid products (μmol)		Conversion (%)
				Acetophenone	Pinacol	
1	+	−	40.93	17.50	17.65	53.36
2	−	−	−	−	−	−
3 ^b^	+	−	−	−	−	−
4 ^c^	+	CCl_4_	1.09	31.40	6.88	47.60
5 ^d^	+	TEOA	3.51	0.70	−	0.84
6 ^e^	+	DMPO	5.98	14.48	−	22.03

^a^ Reaction conditions: 5 mg of photocatalyst, 0.1 mmol of 1-phenylethanol, 5 ml of CH_3_CN, quenchers (0.1 mmol), Ar atmosphere, 2 h. ^b^ Without catalyst. ^c^ CCl_4_ as electron sacrificial agent for capturing photogenerated electrons. ^d^ TEOA as hole sacrificial agent for trapping photogenerated holes. ^e^ DMPO as radical scavenger. + means with light irradiation. − means without corresponding condition or product.

The above results verified the successful construction of heterostructured ZnS-CdS hybrid with close heterointerface contacts and efficient electrons and holes separation, and then we move to identify the bandgap positions of CdS and ZnS QDs. The intrinsic bandgaps of CdS and ZnS QDs are obtained by the Kubelka-Munk function [24], to be 2.52 and 3.65 eV, respectively (Fig. 5C). Determined from the Mott-Schottky plots (Fig. 5D), the flat band potential, which refers to the CB of CdS and ZnS QDs, is calculated to be −0.96 and −1.23 V (vs. Ag/AgCl), respectively. On the equation between the Ag/AgCl and NHE, *E*_NHE_ = *E*_Ag/AgCl_ + 0.197 V [17], the CB values of CdS and ZnS QDs are estimated to be −0.76 and −1.03 V vs. NHE, respectively. Consequently, the VB values of CdS and ZnS QDs are 1.76 and 2.62 V vs. NHE, respectively, and the schematic diagram of CdS and ZnS QDs band structures can be seen in Fig. 5E. In addition, the charge carrier density (*N*_D_) is obtained from Mott-Schottky analysis, and both CdS and ZnS QDs have similar CB state density (see the corresponding calculation details in the Supplementary Materials). Consequently, the band structure and state density in different samples are not crucial factors for photocatalytic performance, and the low activities of bare CdS and ZnS QDs are mainly due to the rapid recombination of electron-hole pairs. On the basis of unique straddling energy band structure of CdS and ZnS, a typical type-I charge transfer pathway at the interface between QDs can be envisaged [36,49–51].

On the basis of the above analysis, a possible catalytic reaction mechanism of such type-I heterostructure for photocatalyzed-redox 1-phenylethanol coupling with H_2_ evolution is proposed [52,53]. As portrayed in Fig. 5E, both CdS QDs and ZnS QDs in the hybrid can be photoexcited to create electron-hole pairs when illuminated by UV-vis light. Favorable electric field driving force and the close contact between CdS QDs and ZnS QDs in the assembled aggregates enable the transfer of photogenerated holes from excited ZnS QDs to the VB of CdS QDs owing to the type-I charge transfer manner, and then the Cα–H bond of 1-phenylethanol is oxidized, offering ^•^C(CH_3_)(OH)Ph and protons [35,54]. The intermediates are further oxidized to acetophenone by photogenerated holes or coupled to generate pinacol [47]. Meanwhile, the electrons are accumulated in the CB of the CdS QDs interact with the protons extracted from the Cα–H bond of 1-phenylethanol to form H_2_.

## Conclusion

In summary, we have reported the successful construction of heterostructured ZnS-CdS hybrids via a self-assembled process for coupling photocatalyzed-redox 1-phenylethanol dehydrogenation at ambient temperature and pressure. It has been demonstrated that the self-assembly of typical type-I band-matched heterostructured hybrid with intimate interfacial contact and short charge transfer distance can facilitate photogenerated electrons and holes separation. As a consequence, the ZnS-CdS hybrid delivers the improved photocatalyzed-redox activity and stability, and a 3.5-fold enhancement in product yield is further obtained over ZnS-CdS/SiO_2_ composite by employing the scattered light in the near field of the spherical SiO_2_ support. Notably, the hybrid can also efficiently oxidize a variety of aromatic alcohols, where the ratio of electrons and holes consumed in such cooperative redox catalysis is close to 1.0, showing a stoichiometric reaction. We believe that such ZnS-CdS hybrid heterostructure with suitable redox potential can provide a perspective that electrons and holes are used to convert aromatic alcohols into clean fuels and high-value-added fine chemicals.

## Materials and Methods

### Materials

All chemicals were of analytical grade and used without further purification. Zinc chloride (ZnCl_2_), cadmium chloride hemi(pentahydrate) (CdCl_2_·2.5H_2_O), sodium hydroxide (NaOH), hydrochloric acid (HCl), ammonium hydroxide (NH_3_·H_2_O), absolute ethanol (C_2_H_5_OH), acetonitrile (CH_3_CN), carbon tetrachloride (CCl_4_) and TEOA (C_6_H_15_NO_3_) were obtained from Sinopharm Chemical Reagent Co., Ltd. (Shanghai, China). Sodium sulfide nonahydrate (Na_2_S·9H_2_O), 1-phenylethanol (C_8_H_10_O), 2,3-diphenyl-2,3-butanediol (C_16_H_18_O_2_, pinacol), acetophenone (C_8_H_8_O), MPA (C_3_H_6_O_2_S), isopropyl alcohol (C_3_H_8_O), tetraethyl orthosilicate (C_8_H_20_O_4_Si), BPEI (molecular weight = 25,000), DMPO (C_6_H_11_NO), and *N*, *N*-dimethylformamide (C_3_H_7_NO) were purchased from Sigma-Aldrich. Deionized (DI) water was obtained from local sources.

### Synthesis of CdS QDs

The synthetic method of CdS QDs was consistent with the literature [16], and the detailed preparation procedures were described in the Supplementary Materials.

### Synthesis of ZnS QDs

The preparation steps of ZnS QDs took the same method of CdS QDs with minor modifications. Briefly, 1 mmol of ZnCl_2_ was dissolved in 20 ml of DI water followed by 1.7 mmol of MPA. The pH of the solution was controlled to about 10 by dropping NaOH solution (5 M) and then transferred into a 50-ml 3-necked flask and purged with argon (Ar) to expel the air inside. Five milliliters of Na_2_S·9H_2_O solution (0.2 M) was injected into the stirred solution through a syringe, and the solution turned yellow and transparent. Next, the reaction mixture with a condenser was heated to 100 °C and stirred for 4 h. After cooling, C_2_H_5_OH was added to precipitate the separation products, and finally, the synthesized ZnS QDs were preserved in water.

### Synthesis of ZnS-CdS hybrids

The ZnS-CdS hybrids were synthesized by self-assembly method [23]. In detail, a certain amount of ZnS QDs was added to an aqueous solution containing 100 mg of CdS QDs, and then HCl (0.1 M) was added to control the pH to 3. In this process, the surface ligands were separated from the QDs to form self-assembled aggregates, and the solution changed from transparent yellow to turbid yellow. Finally, the as-prepared ZnS-CdS hybrids were washed by DI water at least 3 times and placed in the oven under 60 °C overnight. The obtained hybrids with different weight contents of ZnS (1%, 3%, 5%, 7%, and 9%) were labeled as 1%ZnS-CdS, 3%ZnS-CdS, 5%ZnS-CdS, 7%ZnS-CdS, and 9%ZnS-CdS, respectively.

### Synthesis of ZnS-CdS/SiO_2_

Briefly, 0.2 g of BPEI-SiO_2_ was ultrasonically dispersed in 200 ml of DI water. Subsequently, a proportion of CdS QDs and ZnS QDs were added to the dispersion and placed in a 60 °C oil bath. After stirring for 5 min, HCl (0.1 M) was added to adjust the pH to 3 and stirring for another 1 h. Finally, the obtained ternary composites were washed by DI water and placed in the oven under 60 °C overnight. The as-prepared sample was defined as ZnS-CdS/SiO_2_, and the loading weight content of ZnS-CdS on SiO_2_ spheres was 2.5%.

### Photocatalytic performance testing

Photocatalytic oxidation of 1-phenylethanol integrated with hydrogen (H_2_) evolution was conducted in a 40-ml sealed quartz reactor [42]. In a typical experiment, 5 mg of catalyst was dispersed in 5 ml of CH_3_CN containing 1-phenylethanol (0.1 mmol). The solution was purged with Ar for 20 min to expel the air inside and then illuminated with UV-vis light (300 nm ≤ λ ≤ 800 nm) through a 300-W Xe arc lamp (PLS-SXE 300D, Beijing Perfectlight Co., Ltd) for 2 h at room temperature. The incident light intensity was 300 mW·cm^−2^ which was identified by a PL-MW2000 photoradiometer (Beijing Perfectlight Co., Ltd). The release of H_2_ was quantified via a gas chromatograph (Shimadzu GC-2014C, 5A column, Ar carrier). The liquid products were analyzed by HPLC (Shimadzu HPLC-LC20AT, C18 column) and GC-MS (Shimadzu GC-MS QP 2020, Q-Exactive).

## Data Availability

All data needed to evaluate the conclusions in the paper are present in the paper and the Supplementary Materials. Additional data related to this paper may be requested from the authors.

## Supplementary Materials

Supplementary Materials Fig. S1. Schematic diagram of MPA ligand-capped (A) CdS QDs and (B) ZnS QDs. Fig. S2. Size distribution of (A) CdS QDs and (B) ZnS QDs measured by DLS. Fig. S3. (A) TEM image of ZnS QDs. (B) HRTEM image and (C) selected-area electron diffraction (SAED) of ZnS-CdS hybrid. Fig. S4. (A) EDX spectrum and (B) element mapping of ZnS-CdS hybrid. Fig. S5. XPS spectra of samples: (A) Survey spectra and (B) high-resolution XPS spectra of C 1s for CdS QDs, ZnS QDs, and ZnS-CdS hybrid. Fig. S6. Mass spectra of 1-phenylethanol (feedstock) and obtained liquid products (acetophenone and pinacol). Fig. S7. (A to C) HPLC analytical chromatograms of different concentrations of 1-phenylethanol, acetophenone, and pinacol. (D to F) Peak areas corresponding to known product concentrations were used for the calibration curves. Fig. S8. (A) XRD patterns and (B) DRS spectra of fresh and used 5%ZnS-CdS hybrid. Fig. S9. (A) HRTEM image and (B) high-angle annular dark field and corresponding elemental mapping results of used ZnS-CdS hybrid. Fig. S10. Recycling photocatalytic activity tests over bare CdS QDs. Fig. S11. (A) TEM image, (B) HRTEM image, and (C) high-angle annular dark field and corresponding elemental mapping results of ZnS-CdS/SiO_2_. Fig. S12. DRS spectra of SiO_2_, BPEI-SiO_2_, 5%ZnS-CdS, and 5%ZnS-CdS/SiO_2_. Fig. S13. Photocatalytic performance over bare CdS QDs, 5%ZnS-CdS, and 5%ZnS-CdS/SiO_2_. Fig. S14. Mass spectra of benzyl alcohol (feedstock) and obtained liquid products (benzaldehyde and hydrobenzoin). Fig. S15. Mass spectra of 4-methylbenzyl alcohol (feedstock) and obtained liquid products (4-methylbenzaldehyde and 1,2-di-p-tolylethane-1,2-diol). Fig. S16. Mass spectra of 4-methoxybenzyl alcohol (feedstock) and obtained liquid product (4-methoxybenzaldehyde). Fig. S17. Mass spectra of 4-chlorobenzyl alcohol (feedstock) and obtained liquid products (4-chlorobenzaldehyde and 1,2-bis(4-chlorophenyl)ethane-1,2-diol). Fig. S18. Mass spectra of 4-nitrobenzyl alcohol (feedstock) and obtained liquid product (p-nitrobenzaldehyde). Fig. S19. UV-vis absorption spectroscopy and steady-state spectroscopy of (A) CdS QDs and (B) ZnS QDs. Fig. S20. LC-MS chromatograph of reaction system with DMPO as the radicals-trapping agent upon 2-h irradiation. Table S1. A summary on the parameters for XPS peaks of C1s in Fig. S5B. Table S2. The kinetic analysis of emission decay for bare CdS QDs, bare ZnS QDs, and 5%ZnS-CdS hybrid.
