# Supplementary material for: Heterostructure-Engineered Semiconductor Quantum Dots toward Photocatalyzed-Redox Cooperative Coupling Reaction
Source: Research (Wash D C). 2023 Mar 10;6:0073. doi: 10.34133/research.0073 (PMC10013965; doi:10.34133/research.0073)
Supplement: Supplementary Materials — Fig. S1. Schematic diagram of MPA ligand-capped (A) CdS QDs and (B) ZnS QDs. Fig. S2. Size distribution of (A) CdS QDs and (B) ZnS QDs measured by DLS. Fig. S3. (A) TEM image of ZnS QDs. (B) HRTEM image and (C) selected-area electron diffraction (SAED) of ZnS-CdS hybrid. Fig. S4. (A) EDX spectrum and (B) element mapping of ZnS-CdS hybrid. Fig. S5. XPS spectra of samples: (A) Survey spectra and (B) high-resolution XPS spectra of C 1s for CdS QDs, ZnS QDs, and ZnS-CdS hybrid. Fig. S6. Mass spectra of 1-phenylethanol (feedstock) and obtained liquid products (acetophenone and pinacol). Fig. S7. (A to C) HPLC analytical chromatograms of different concentrations of 1-phenylethanol, acetophenone, and pinacol. (D to F) Peak areas corresponding to known product concentrations were used for the calibration curves. Fig. S8. (A) XRD patterns and (B) DRS spectra of fresh and used 5%ZnS-CdS hybrid. Fig. S9. (A) HRTEM image and (B) high-angle annular dark field and corresponding elemental mapping results of used ZnS-CdS hybrid. Fig. S10. Recycling photocatalytic activity tests over bare CdS QDs. Fig. S11. (A) TEM image, (B) HRTEM image, and (C) high-angle annular dark field and corresponding elemental mapping results of ZnS-CdS/SiO2. Fig. S12. DRS spectra of SiO2, BPEI-SiO2, 5%ZnS-CdS, and 5%ZnS-CdS/SiO2. Fig. S13. Photocatalytic performance over bare CdS QDs, 5%ZnS-CdS, and 5%ZnS-CdS/SiO2. Fig. S14. Mass spectra of benzyl alcohol (feedstock) and obtained liquid products (benzaldehyde and hydrobenzoin). Fig. S15. Mass spectra of 4-methylbenzyl alcohol (feedstock) and obtained liquid products (4-methylbenzaldehyde and 1,2-di-p-tolylethane-1,2-diol). Fig. S16. Mass spectra of 4-methoxybenzyl alcohol (feedstock) and obtained liquid product (4-methoxybenzaldehyde). Fig. S17. Mass spectra of 4-chlorobenzyl alcohol (feedstock) and obtained liquid products (4-chlorobenzaldehyde and 1,2-bis(4-chlorophenyl)ethane-1,2-diol). Fig. S18. Mass spectra of 4-nitrobenzyl alcohol (feedstock) and ob [file research.0073.f1.pdf]

Supporting Information

Heterostructure Engineered Semiconductor Quantum Dots toward Photocatalyzed-Redox Cooperative Coupling Reaction

Lin-Xing Zhang, Ming-Yu Qi, Zi-Rong Tang*, and Yi-Jun Xu*

College of Chemistry, State Key Laboratory of Photocatalysis on Energy and Environment, Fuzhou University, Fuzhou, 350116, P.R. China

Correspondence should be addressed to Zi-Rong Tang; zrtang@fzu.edu.cn, and Yi-Jun Xu; yjxu@fzu.edu.cn

Experimental procedures

Synthesis of CdS QDs: Typically, 1 mmol of $CdCl_2·2.5H_2O$ was dissolved in 20 mL of DI water, and then 1.7 mmol of MPA was added and the pH was controlled to 10 by adding NaOH solution (5 M). The solution was transferred into a 50 mL three-necked flask and purged with Ar to expel the air inside. Under magnetic stirring, 5 mL of $Na_2S·9H_2O$ solution (0.2 M) was injected through a syringe. Subsequently, the reaction mixture was heated to 100 ℃, equipped with a condenser, and the solution was stirred for 0.5 h to promote the growth of QDs. The white transparent solution was cooled and C_2H_5OH was added to separate the product and then preserved in water.

Synthesis of SiO_2 spheres: SiO_2 spheres were synthesized *via* the Stöber method. Briefly, 2 mL of TEOS was added into a mixture of 40 mL of isopropanol, 1 mL of DI water, and 2 mL of $NH_3·H_2O$ (10.5 M) and stir for 4 h. Subsequently, the colloidal spheres were washed with C_2H_5OH and DI water and placed in the oven under 60 ℃ overnight. To obtain SiO_2 spheres with positive surface charges, BPEI was used for surface functionalization. 400 mg of SiO_2 was dispersed in 200 mL of C_2H_5OH by ultrasound and then 344 mg of BPEI was added. After stirring at 60 ℃ for 4 h, BPEI-SiO_2 spheres were washed with C_2H_5OH and DI water, and dried at 60 ℃ overnight.

Characterization: The morphology and distribution of samples were analyzed by transmission electron microscopy (TEM), high-resolution TEM (HRTEM), energy dispersive X-ray spectrum (EDX) and elemental mapping analysis using a FEI Talos F200X instrument at an accelerating voltage of 200 kV. The phase composition of the sample was measured by X-ray diffraction (XRD) on a Rigaku Miniflex diffractometer with Cu Kα radiation in the 2θ range from 10 to 80 ° at a scan rate of 0.02 °/s. Ultraviolet-visible diffuse reflectance spectroscopy (DRS) was used to measure the light absorption characteristics of the sample on an UV-vis spectrophotometers (Thermo Scientific Evolution 200 Series), with $BaSO_4$ as a reflectance standard. X-ray photoelectron spectroscopy (XPS) was used to analyze the chemical composition and valence states of the sample surface. (Thermo Scientific K-Alpha). All binding energies were calibrated at 284.80 eV using the C 1s peak. Fourier transform infrared spectroscopy (FTIR) was performed on a Nicolet iS50 FT-IR spectrophotometer with a resolution of 4 cm^{-1}. Dynamic light scattering (DLS) was used to measure the particle size of QDs (Zetasizer Nano-ZS90). Photoluminescence (PL) spectra and time-resolved PL (TRPL) decay plots of the samples were analyzed on an Edinburgh Analytical Instrument F900 spectrophotometer. Electron paramagnetic resonance (EPR) spectroscopic measurements were performed at room temperature using a Bruker A300 EPR spectrometer. For EPR measurements, 5 mg of sample was dispersed in a mixed solution of 5 mL of CH_3CN containing 1-phenylethanol (0.1 mmol) and DMPO (0.1 mmol) by ultrasonic treatment. Then, the suspension was injected into a glass capillary tube, and placed in a sealed glass tube under an Ar atmosphere, and then irradiated with 300 W Xe arc lamp at room temperature for EPR testing. Reaction intermediates were examined by liquid chromatography mass spectrometry (LC-MS, Thermo Scientific Exactive Plus).

Photoelectrochemical measurements: The photoelectrochemical and electrochemical measurements were carried out in a standard three-electrode cell, using a Pt sheet as the counter electrode, and a saturated Ag/AgCl electrode as the reference electrode, and the working

electrode was fluorine doped tin oxide (FTO) glass. Firstly, the FTO glass is ultrasonic cleaned with ethanol and DI water. After drying, using transparent tape to protect the boundary of the FTO glass. Secondly, the 5 mg of sample was completely dispersed in a mixed solvent consisting of 0.5 mL of DMF and 50 μL of membrane solution by ultrasonic treatment to obtain a slurry and the slurry (20 μL) was coated on the pre-treated FTO glass. After air drying, the working electrode was further dried at 60 ℃ for 1 h to improve adhesion. The exposed area of the working electrode was 0.25 cm^2. The transient photocurrent measurement was performed in 0.2 M Na_2SO_4 aqueous solution (pH = 6.8) without voltage bias under the UV-vis light irradiation. The electrochemical impedance spectroscopy (EIS), polarization curves and cyclic voltammetry (CV) curves were measured by the electrochemical work station (MUTI AUTOLAB M204) in the three-electrode battery. Mott-Schottky plots were conducted at a frequency of 500 and 1000 Hz with a bias potential ranged from −1 to 2 V *vs.* Ag/AgCl.

Photocatalytic recycling tests: In order to evaluate the photocatalytic stability and recyclability of the catalyst, recycling tests were carried out. The specific experimental steps are as follows. At the end of the first photocatalytic reaction, the catalyst was centrifuged and washed with DI water for three times. After drying, fresh 0.1 mmol of 1-phenylethanol and 5 mL of CH_3CN were added with the used catalyst for the second cycle test. Subsequent recycle tests were conducted in a similar manner. The conversion of 1-phenylethanol was calculated with the following equation:

$$\text{Conversion (\%)} = \frac{n_0 - n_1}{n_0} \times 100\%$$

$$\text{Carbon balance (\%)} = [n(\text{acetophenone}) + 2 \times n(\text{pinacol})] / (n_0 - n_1)$$

where n_0 represents the initial molar amount of 1-phenylethanol; n_1 is the molar amount of 1-phenylethanol remaining after the catalytic reaction.

Determination of apparent quantum yield (AQY): The AQY of H_2 for the 5%ZnS-CdS hybrid was tested under the same reaction conditions, except for the different wavelength monochromatic light irradiation (350, 400, 450, 530 and 600 nm). The irradiation area was fixed to 4.52 cm^2 and taking λ = 400 nm (15.5 mW cm^{-2}) as an example, the details of AQY calculation were as follows [S1].

(i) The number (N_{photon}) of absorbed photons:

$$N_{photon} = \frac{t(s) \times P(W\ cm^{-2}) \times S(cm^{-2}) \times \lambda(m)}{h(J\ s^{-1}) \times c(m\ s^{-1})}$$

$$N_{photon} = \frac{2 \times 3600(s) \times 0.0155(W\ cm^{-2}) \times 4.52(cm^{2}) \times 400 \times 10^{-9}(m)}{6.626 \times 10^{-34}(J\ s^{-1}) \times 3 \times 10^{8}(m\ s^{-1})}$$

$$N_{photon} = 1.015 \times 10^{21}$$

(ii) The AQY is obtained by the following equation:

$$AQY = \frac{2n_{H_2}(mol) \times N_A(mol^{-1})}{N_{photon}} \times 100\%$$

$$AQY = \frac{2 \times 16.11 \times 10^{-6}(mol) \times 6.02 \times 10^{23}(mol^{-1})}{1.015 \times 10^{21}} \times 100\%$$

AQY = 1.91%

Calculation of the band gap energy (E_g): DRS spectra have been employed to identify the band gap positions of CdS and ZnS QDs (**Figure 2c**), and the following formula is used [S2]:

$$(Ah\nu)^n = K\cdot(h\nu - E_g)$$

Where A is the absorption coefficient, hν is the photon energy, K is a constant, E_g is the band gap energy, n is related to the type of semiconductor, CdS and ZnS are direct band gap semiconductor (n = 2). As shown in **Figure 5c**, the E_g of CdS QDs and ZnS QDs are evaluated to be 2.52 and 3.65 eV, respectively.

Calculation of the charge carrier density (N_D): Mott-Schottky analysis (**Figure 5d**) was used to provide fundamental insights into the N_D according to the following equation [S3, S4]:

$$N_D = \frac{2}{e\varepsilon\varepsilon_0}\left[\frac{d(1/C^2)}{dV}\right]^{-1}$$

where e is the elementary electronic charge, ε is the dielectric constant, specifically 6 for CdS and 7 for ZnS [S5], ε_0 is the permittivity in vacuum, C is the capacitance, and V is the applied potential. The N_D of CdS and ZnS are calculated to be $4.19*10^{18}$ and $5.05*10^{18}$ cm^{-3}, respectively, when carried out at a fixed frequency of 1000 Hz.

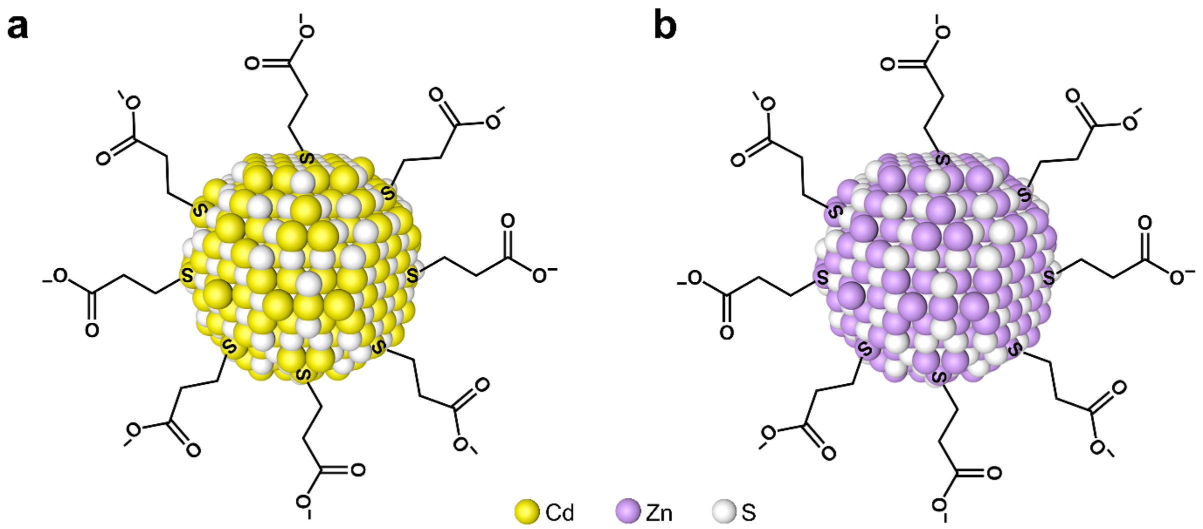

Figure S1. Schematic diagram of MPA ligand capped (a) CdS QDs and (b) ZnS QDs.

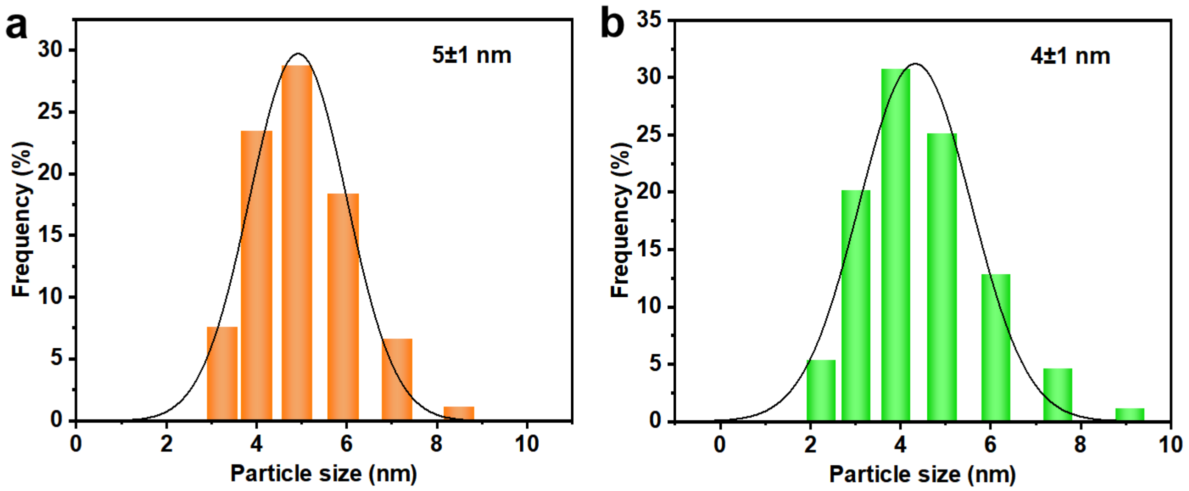

Figure S2. Size distribution of (a) CdS QDs and (b) ZnS QDs measured by DLS.

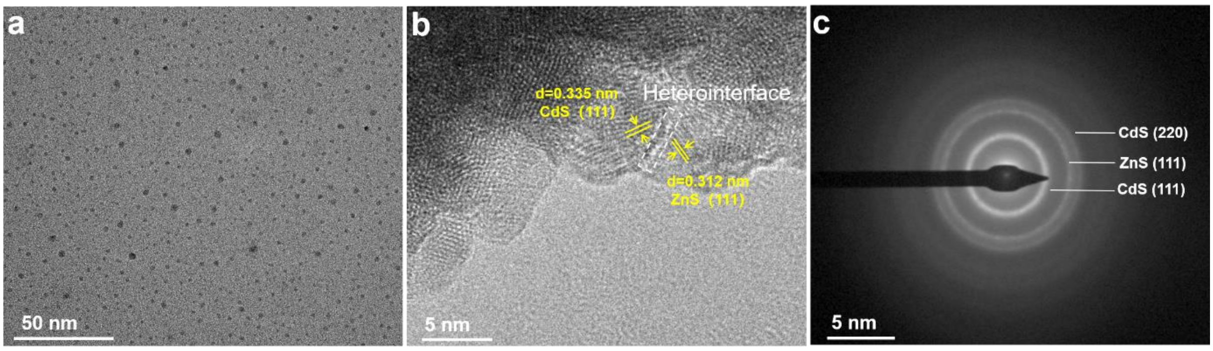

Figure S3. (a) TEM image of ZnS QDs. (b) HRTEM image and (c) selected area electron diffraction (SAED) of ZnS-CdS hybrid.

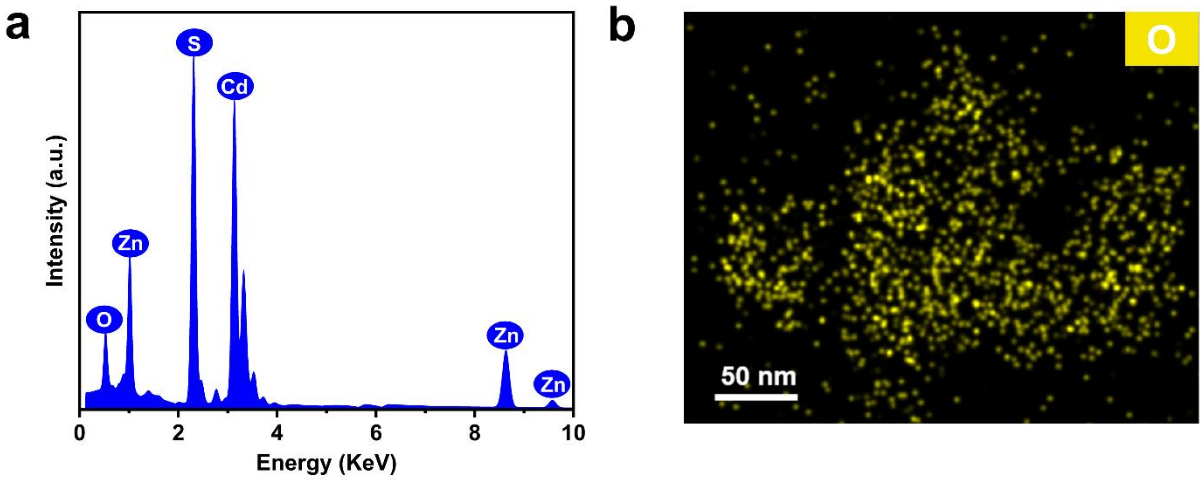

Figure S4. (a) EDX spectrum and (b) element mapping of ZnS-CdS hybrid.

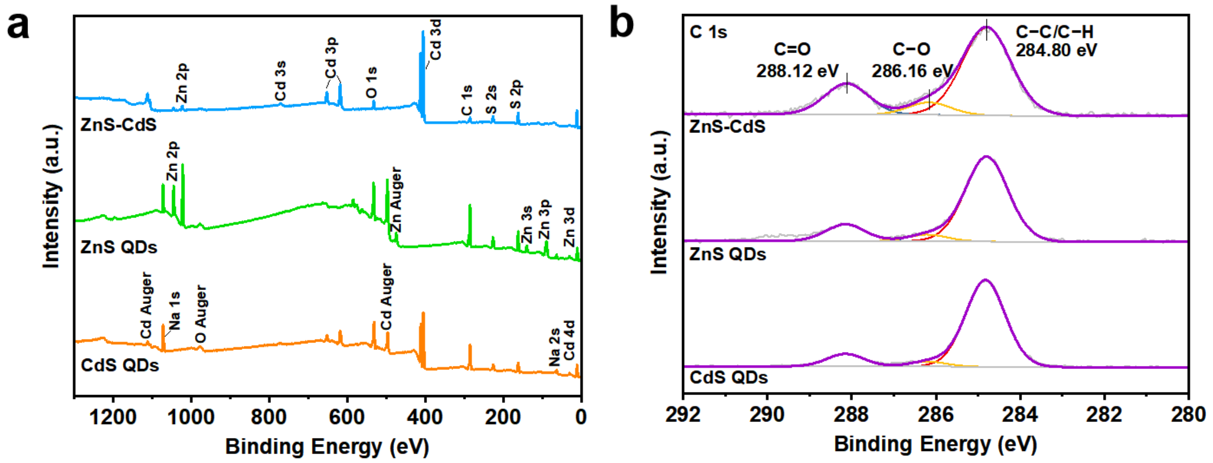

Figure S5. XPS spectra of samples: (a) survey spectra and (b) high-resolution XPS spectra of C 1s for CdS QDs, ZnS QDs and ZnS-CdS hybrid.

Note: In the XPS survey spectra, the Na 1s peak derives from Na_2S in the raw material. In **Figure S5b**, the binding energies of 284.80, 286.16 and 288.12 eV can be assigned to the C−C/C−H bond, C−O bond and C=O bond, respectively [S6]. Based on XPS spectra, the C−C/C−H, C=O and C−O contents of the sample surface can be estimated from the peak areas listed in **Table S1**.

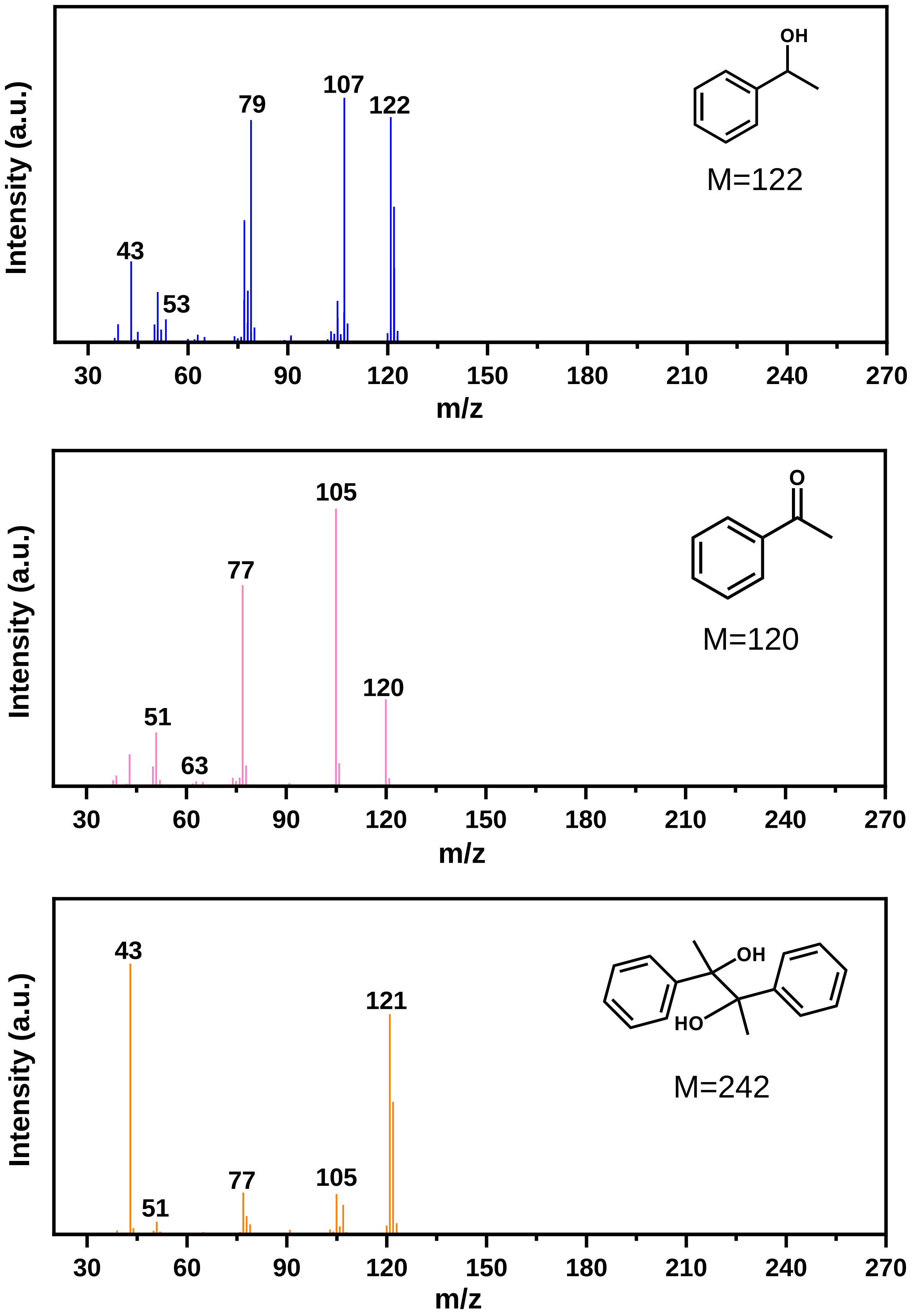

Figure S6. Mass spectra of 1-phenylethanol (feedstock) and obtained liquid products (acetophenone and pinacol).

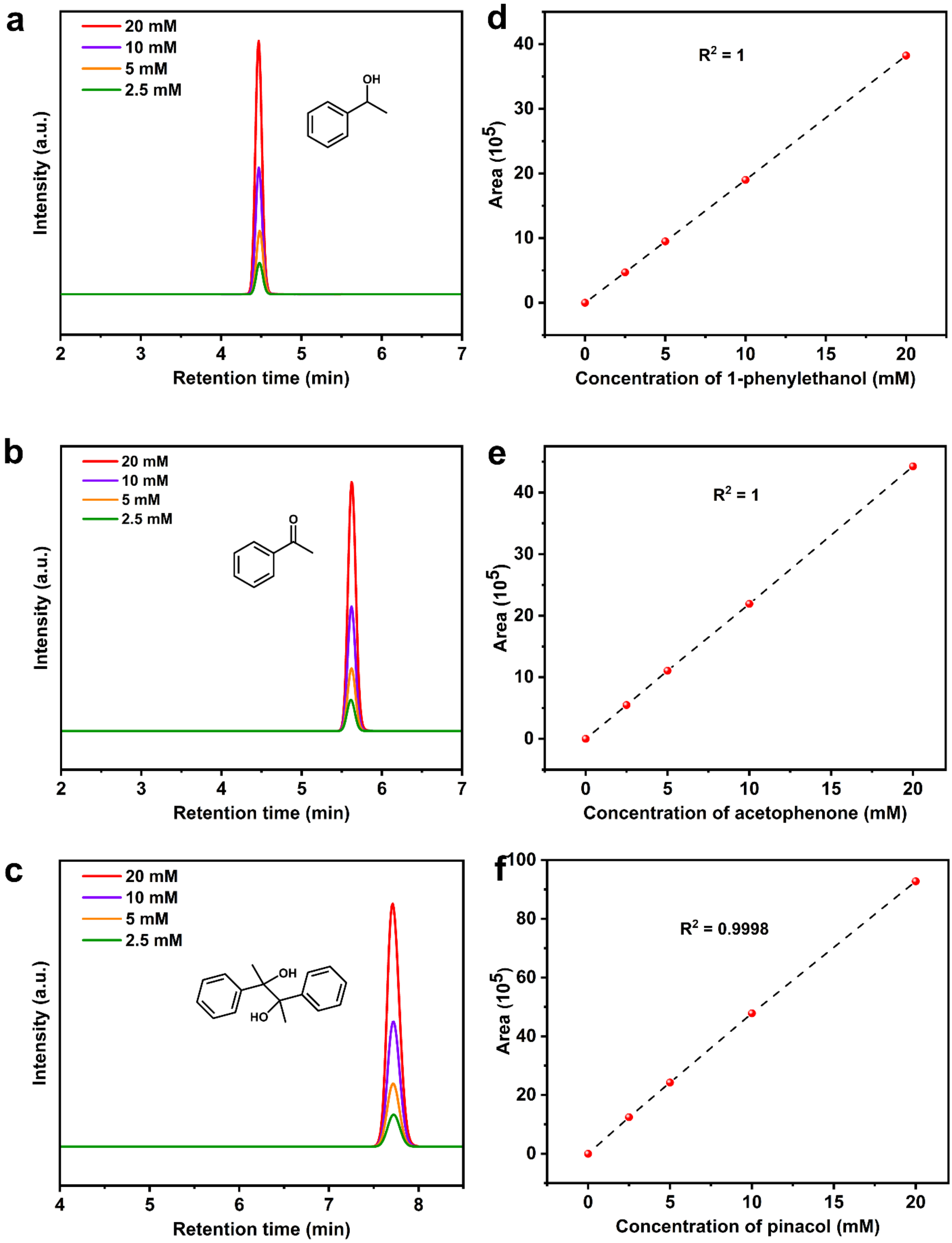

Figure S7. (a-c) HPLC analytical chromatograms of different concentrations of 1-phenylethanol, acetophenone and pinacol. (d-f) Peak areas corresponding to known product concentrations were used for the calibration curves.

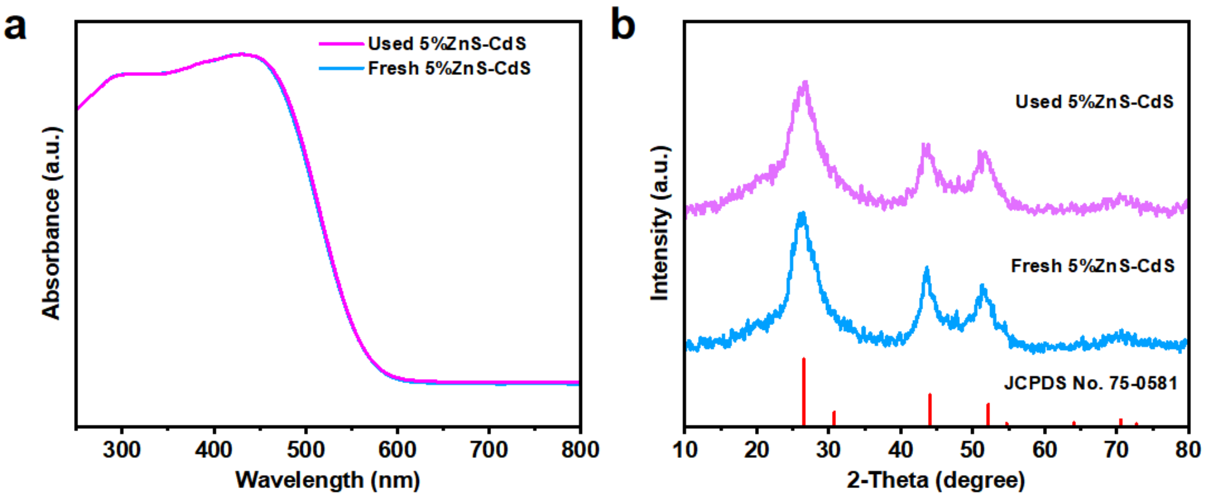

Figure S8. (a) XRD patterns and (b) DRS spectra of fresh and used 5%ZnS-CdS hybrid.

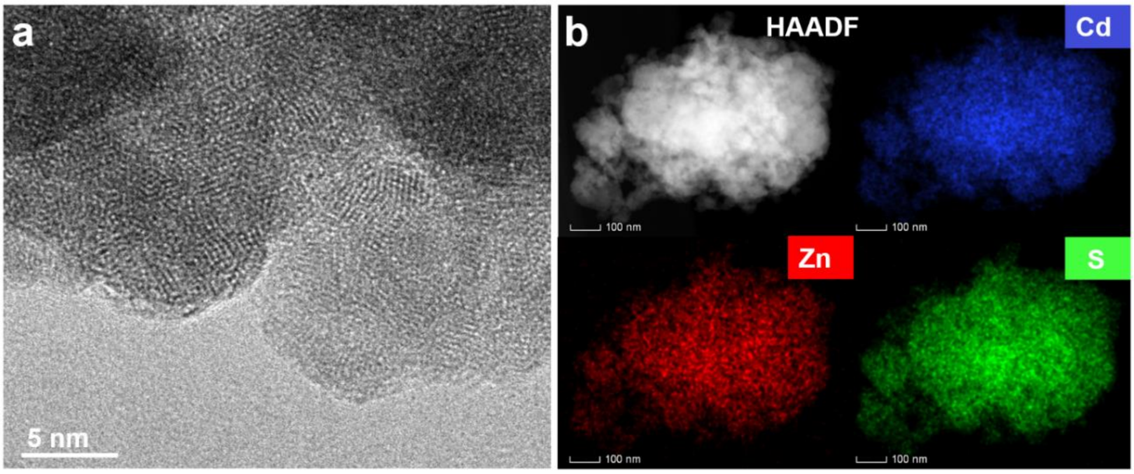

Figure S9. (a) HRTEM image and (b) high-angle annular dark field and corresponding elemental mapping results of used ZnS-CdS hybrid.

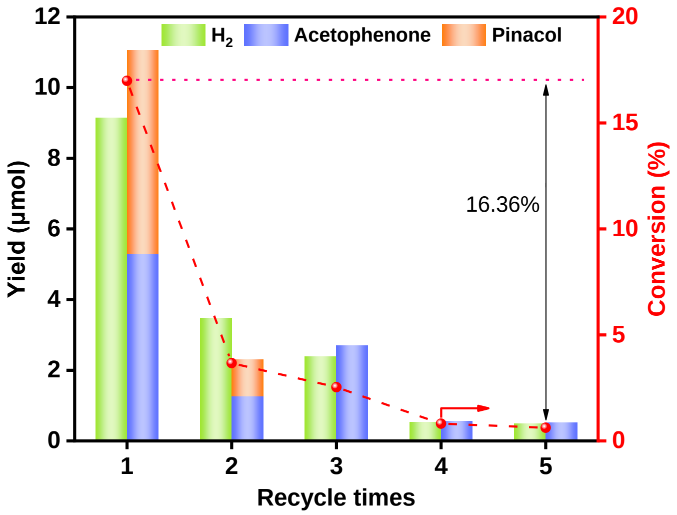

Figure S10. Recycling photocatalytic activity tests over bare CdS QDs.

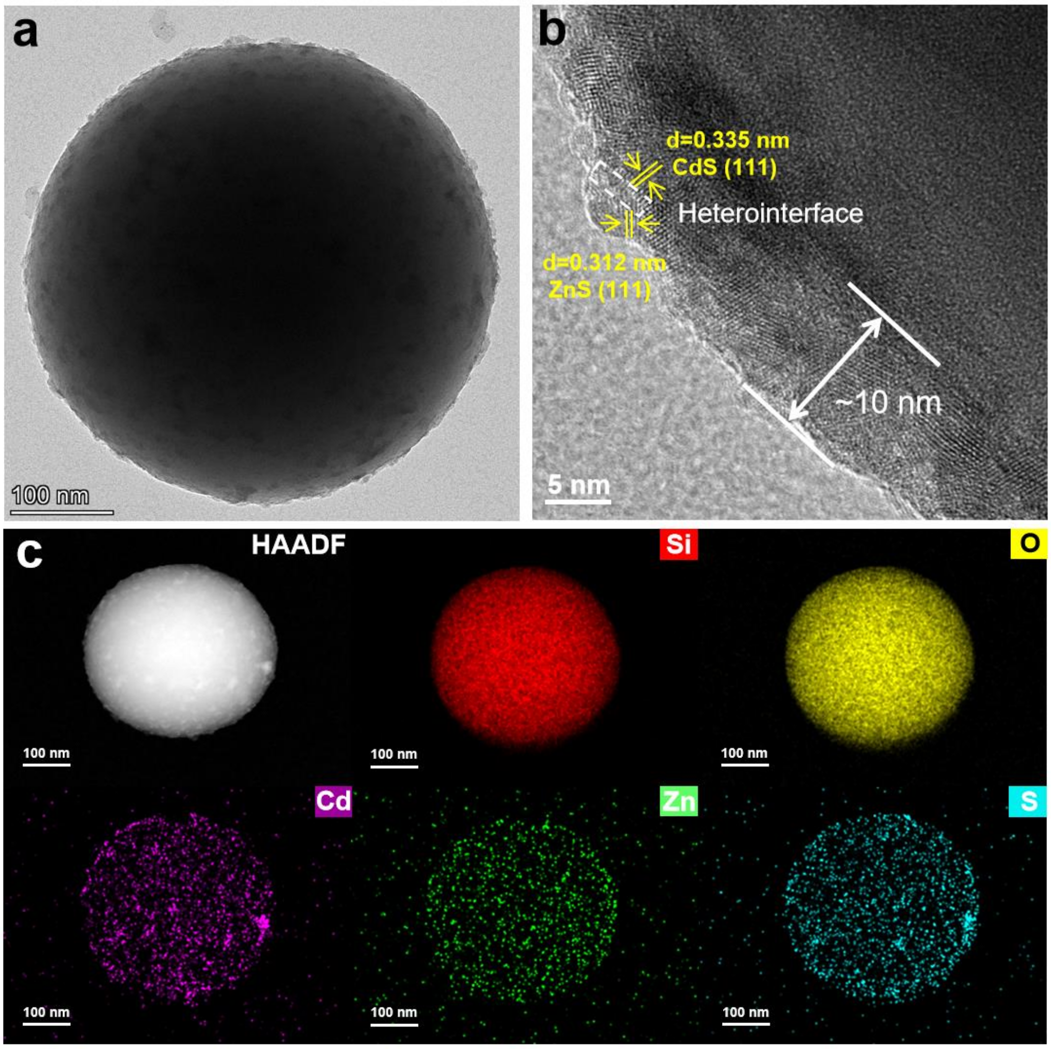

Figure S11. (a) TEM image, (b) HRTEM image and (c) high-angle annular dark field and corresponding elemental mapping results of ZnS-CdS/SiO_2.

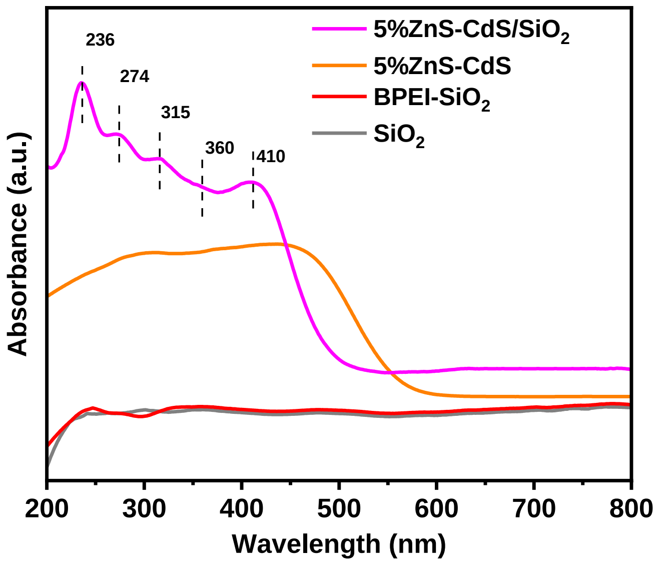

Figure S12. DRS spectra of SiO_2, BPEI-SiO_2, 5%ZnS-CdS and 5%ZnS-CdS/SiO_2.

Note: In order to ensure the quantitative comparability of DRS spectra, $BaSO_4$, serving as an internal reflectance standard, was used as the support and loaded with an equal amount of 5%ZnS-CdS to 5%ZnS-CdS/SiO_2.

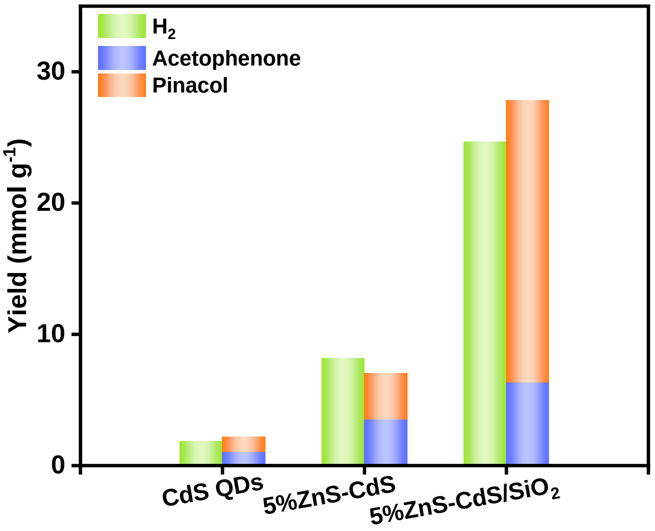

Figure S13. Photocatalytic performance over bare CdS QDs, 5%ZnS-CdS and 5%ZnS-CdS/SiO_2.

Note: Reaction conditions: 10 mg of 5%ZnS-CdS/SiO_2 (including 0.25 mg of 5%ZnS-CdS) was dispersed in 5 mL of CH_3CN, and 0.1 mmol of 1-phenylethanol was added to the solution. Then Ar was purged into the solution for 20 min to expel the air inside and illuminated with 300 W Xe arc lamp (300 nm ≤ λ ≤ 800 nm) for 2 h at room temperature.

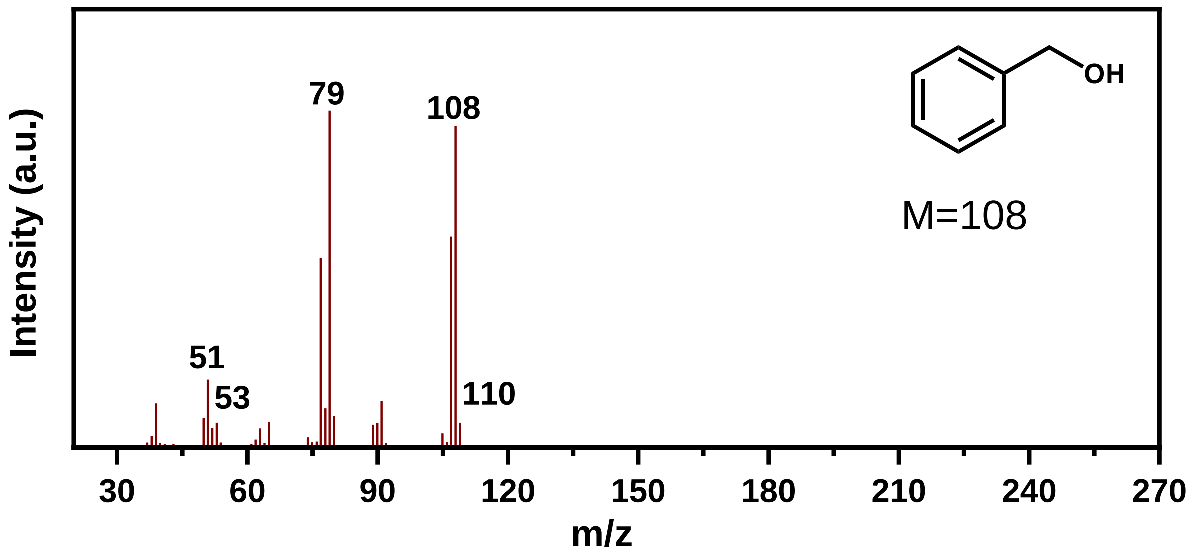
Intensity (a.u.)
79
108
51
53
110
OH
M=108
30
60
90
120
150
180
210
240
270
m/z

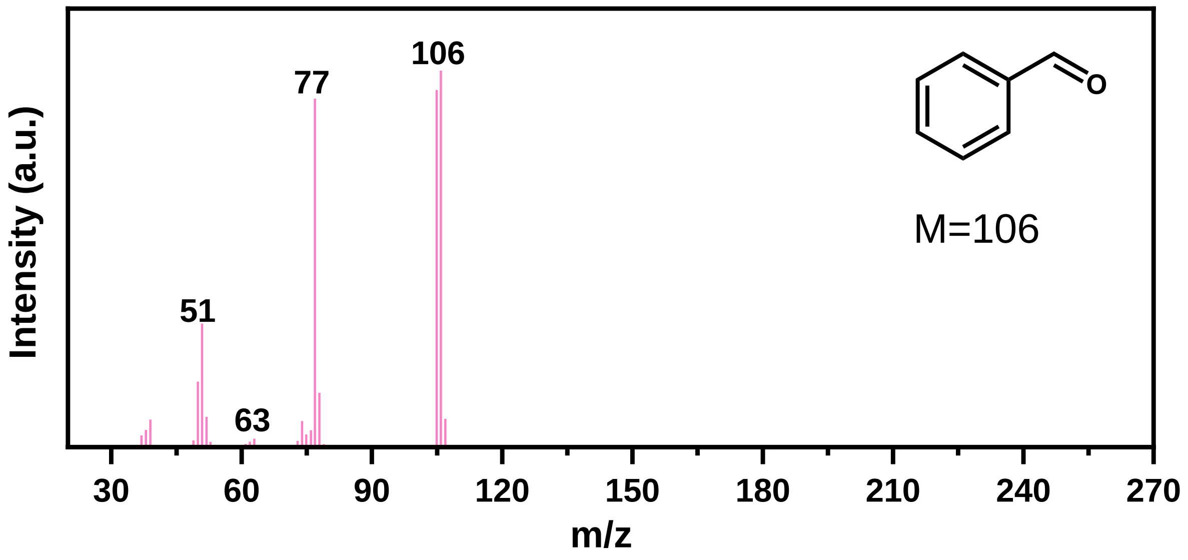
Intensity (a.u.)
106
77
51
63
O
M=106
30
60
90
120
150
180
210
240
270
m/z

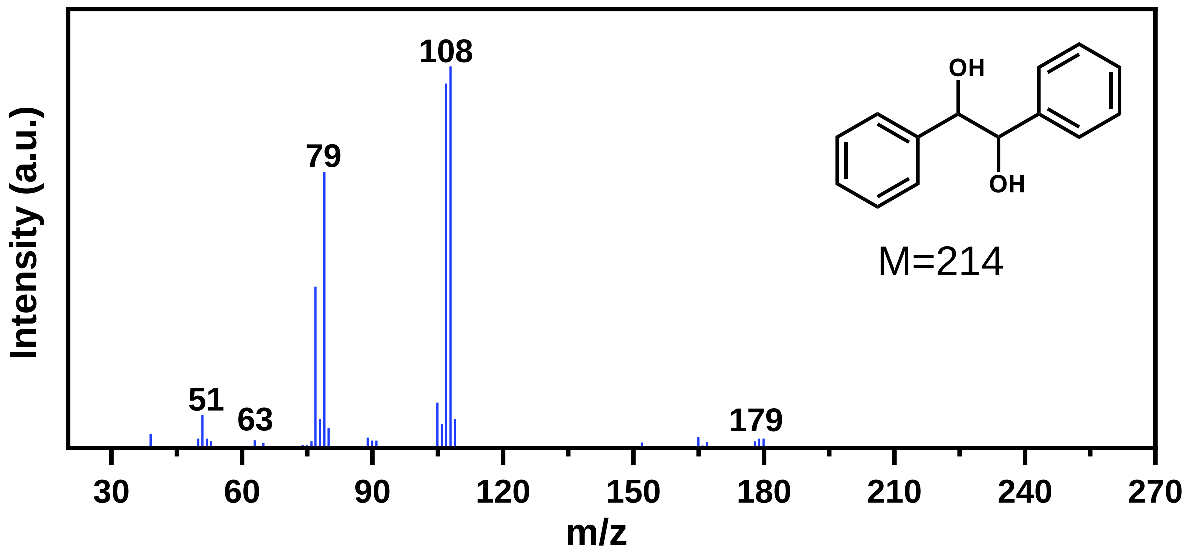
Intensity (a.u.)
108
79
51
63
179
OH
OH
M=214
30
60
90
120
150
180
210
240
270
m/z

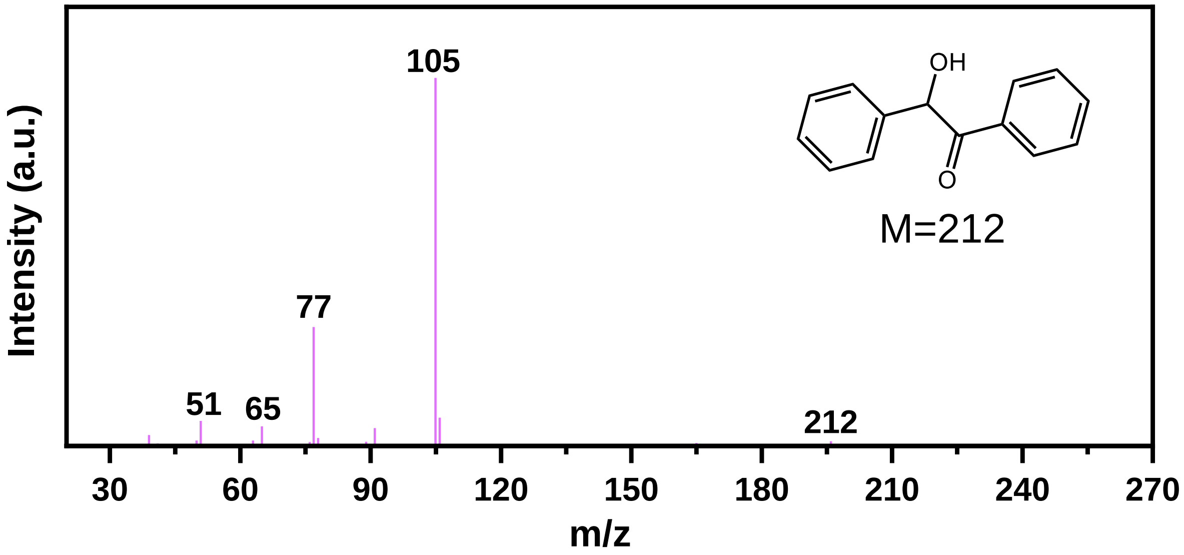

Figure S14. Mass spectra of benzyl alcohol (feedstock) and obtained liquid products (benzaldehyde, hydrobenzoin and benzoin).

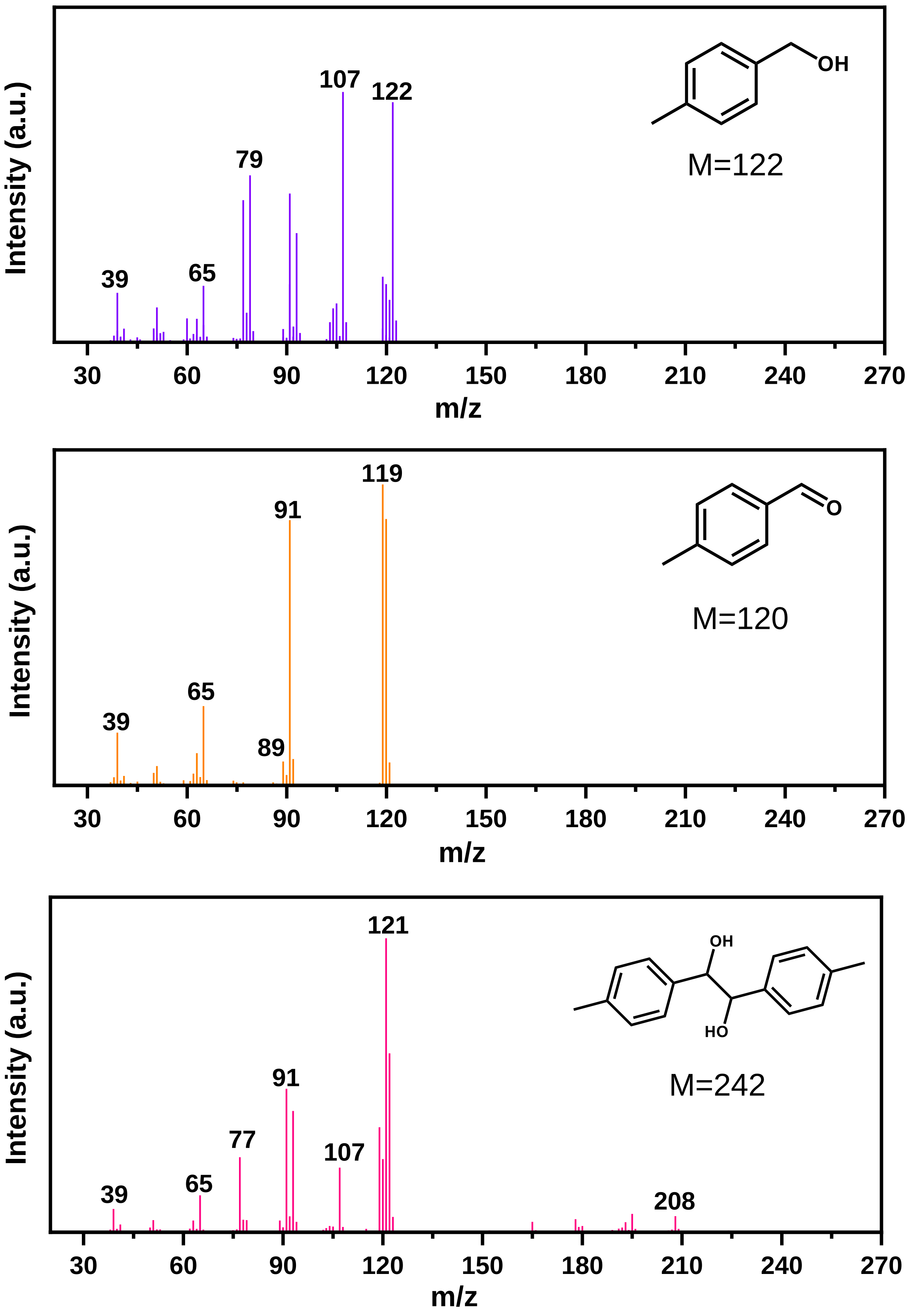

Figure S15. Mass spectra of 4-methylbenzyl alcohol (feedstock) and obtained liquid products (4-methylbenzaldehyde and 1,2-di-p-tolylethane-1,2-diol).

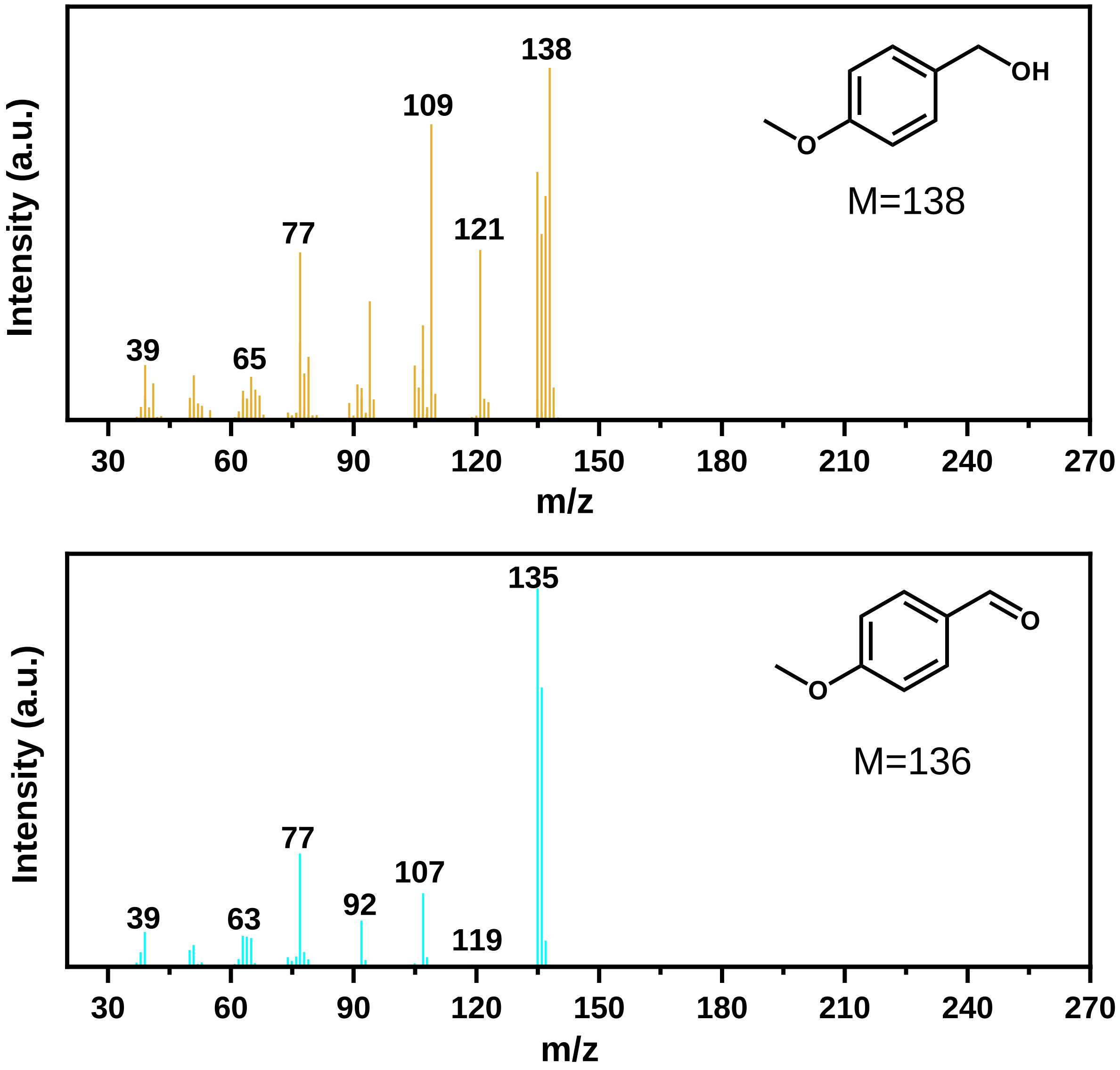

Figure S16. Mass spectra of 4-methoxybenzyl alcohol (feedstock) and obtained liquid product (4-methoxybenzaldehyde).

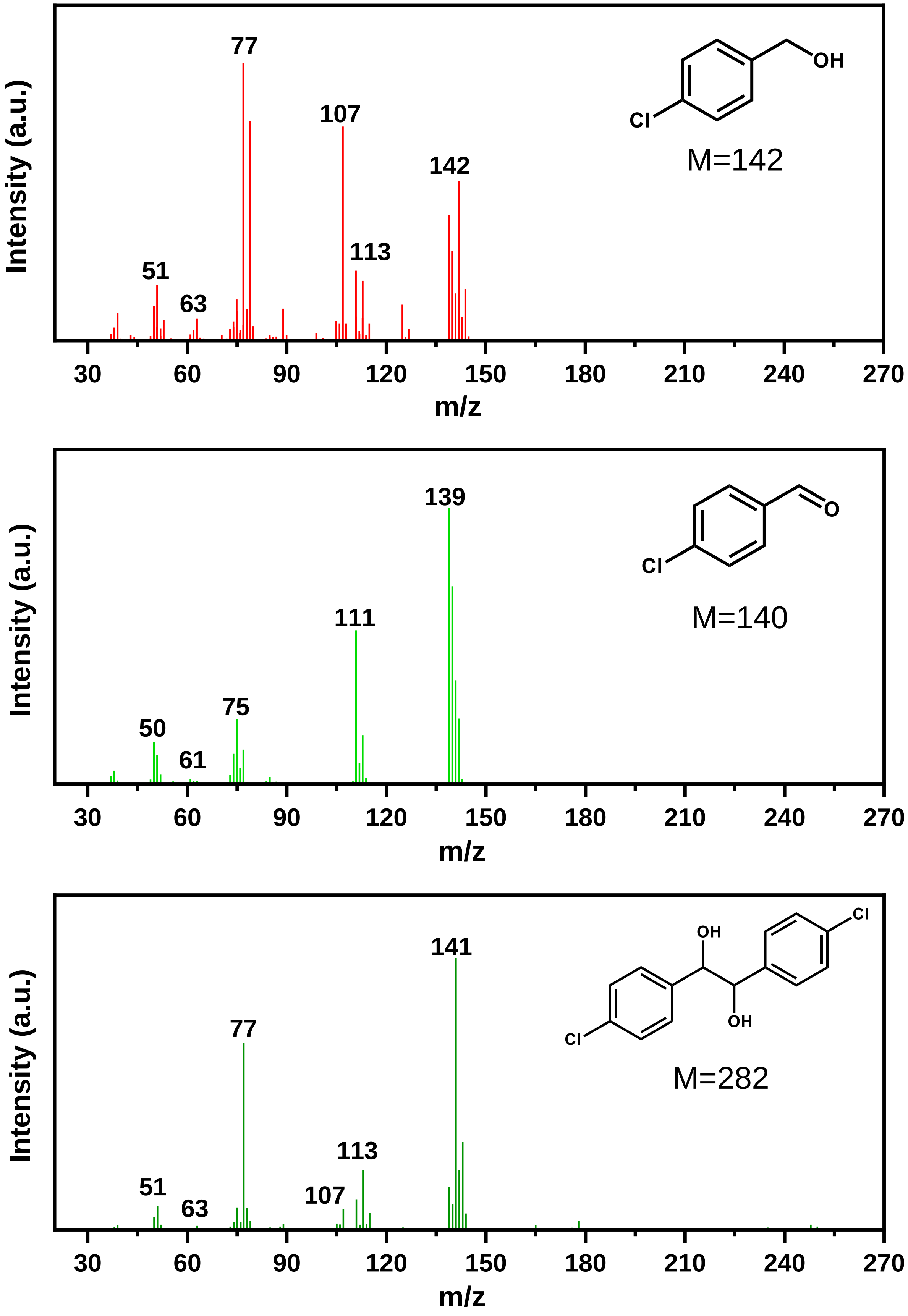

Figure S17. Mass spectra of 4-chlorobenzyl alcohol (feedstock) and obtained liquid products (4-chlorobenzaldehyde and 1,2-bis(4-chlorophenyl)ethane-1,2-diol).

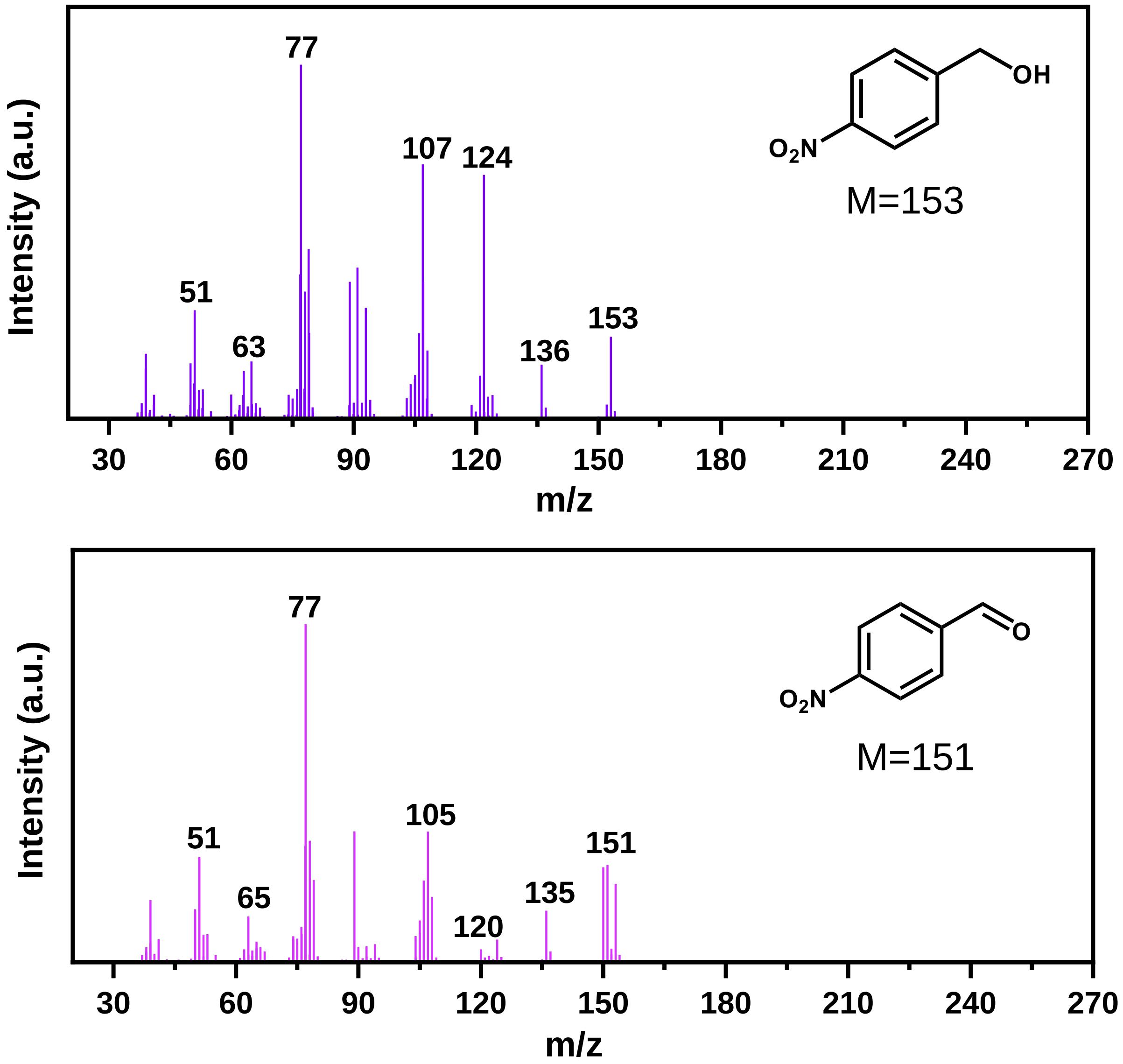

Figure S18. Mass spectra of 4-nitrobenzyl alcohol (feedstock) and obtained liquid product (p-nitrobenzaldehyde).

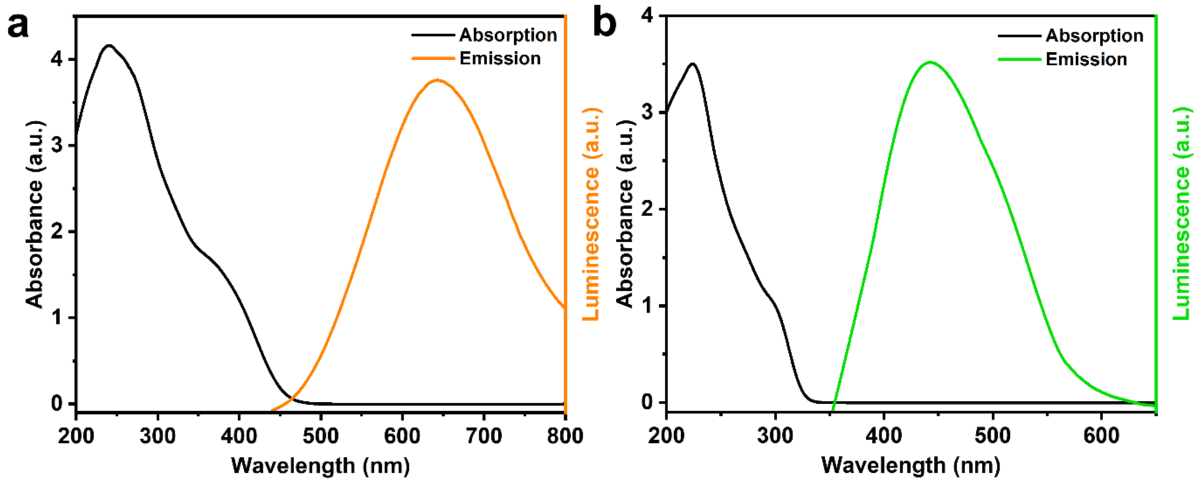

Figure S19. UV-vis absorption spectroscopy and steady-state spectroscopy of (a) CdS QDs and (b) ZnS QDs.

Note: An aqueous dispersion of CdS QDs shows broadband optical absorption from the ultraviolet to the visible-light region with an emission wavelength of about 640 nm (380 nm excitation) [S7]. The absorption of ZnS QDs is below 330 nm and the emission is approximately 450 nm (335 nm excitation) [S8].

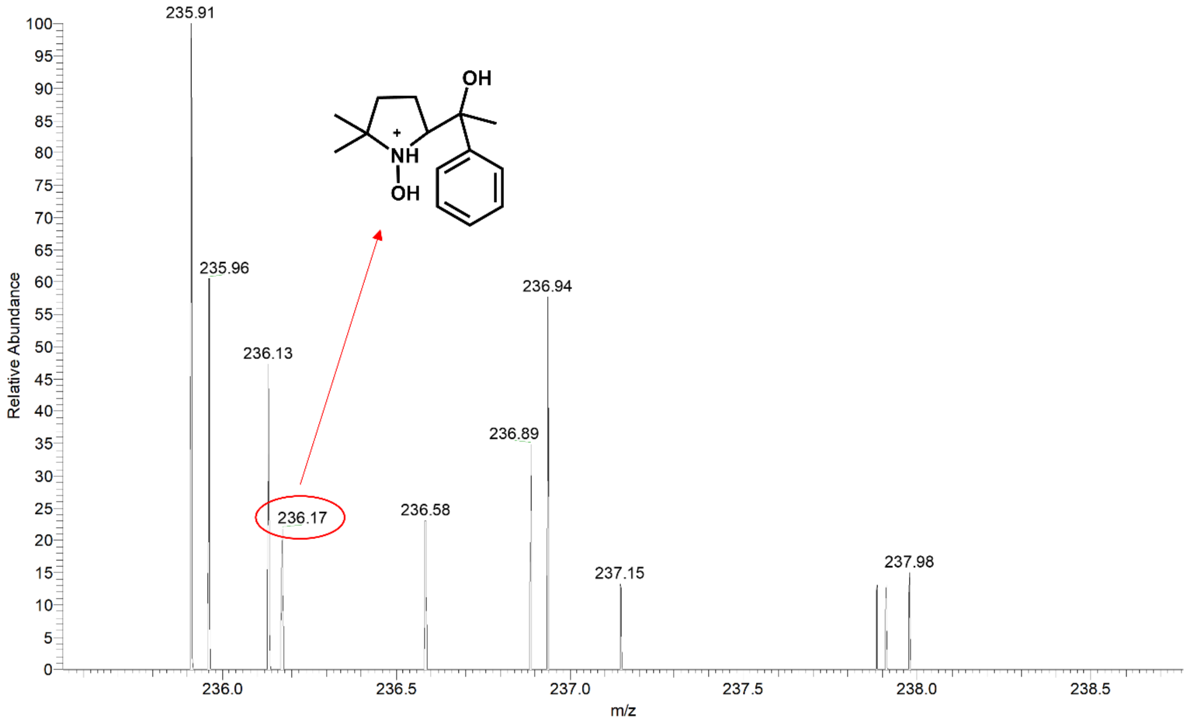

Figure S20. LC-MS chromatograph of reaction system with DMPO as the radicals trapping agent upon 2 h irradiation.

Note: The molecular weight of DMPO and intermediate Cα radical with addition of a H atom (m/z = 236.17) was detected in the mass spectrum, indicating the formation of Cα radical [S1].

Table S1. A summary on the parameters for XPS peaks of C1s in **Figure S5b**.

Samples		C 1s		
		C−C/C−H	C−O	C=O
ZnS-CdS	B.E. (eV)	284.80	286.16	288.12
	Area	8741.65	1070.81	2732.05
ZnS QDs	B.E. (eV)	284.80	286.14	288.16
	Area	11898.78	1236.59	2825.36
CdS QDs	B.E. (eV)	284.82	286.17	288.15
	Area	25743.23	1375.33	3452.67

Note: Where B.E. represents binding energy. Compared with the bare CdS and ZnS QDs, the peak areas of C−C/C−H, C=O, and C−O in C 1s of the hybrid are all decreased, which is caused by the partial detachment of the surface ligand $-SCH_2CH_2COO^-$. This result is consistent with FTIR (**Figure 2a**).

Table S2. The kinetic analysis of emission decay for bare CdS QDs, bare ZnS QDs and 5%ZnS-CdS hybrid.

Catalyst	A_1 (%)	τ_1 (ns)	A_2 (%)	τ_2 (ns)	τ_a (ns)
CdS QDs	18.80	1.59	0.19	13.00	2.46
ZnS QDs	0.22	21.80	22.55	1.47	4.07
5%ZnS-CdS	0.69	4.98	98.38	1.10	1.22

Note: TRPL spectra decay curves were fitted by exponential decay kinetics functions and the average emission lifetime (τ_a) reflecting the overall emission decay behavior of the sample was calculated according to the following equation [S9]:

$$I(t) = A_1\exp(-t/\tau_1) + A_2\exp(-t/\tau_2)$$

$$\tau_a = \frac{A_1\tau_1^2 + A_2\tau_2^2}{A_1\tau_1 + A_2\tau_2}$$

where τ_1 and τ_2 are the emission lifetimes, while A_1 and A_2 are the corresponding amplitudes.

References

[S1] Q. Guo, F. Liang, X.-B. Li et al., "Efficient and selective CO_2 reduction integrated with organic synthesis by solar energy," Chem, vol. 5, no. 10, pp. 2605–2616, 2019.

[S2] M.-Y. Qi, Y.-H. Li, M. Anpo et al., "Efficient photoredox-mediated C–C coupling organic synthesis and hydrogen production over engineered semiconductor quantum dots," ACS Catalysis, vol. 10, no. 23, pp. 14327–14335, 2020.

[S3] Z. Xu, Y. Lin, M. Yin et al., "Understanding the enhancement mechanisms of surface plasmon-mediated photoelectrochemical electrodes: a case study on Au nanoparticle decorated TiO_2 nanotubes," Advanced Materials Interfaces, vol. 2, no. 13, pp. 1500169, 2015.

[S4] N. Zhang, M.-Y. Qi, L. Yuan et al., "Broadband light harvesting and unidirectional electron flow for efficient electron accumulation for hydrogen generation," Angewandte Chemie International Edition, vol. 58, no. 29, pp. 10003–10007, 2019.

[S5] M. Goyal, "A model to determine variation in dielectric constant with size and composition in semiconducting nanosolids," Journal of Computational Electronics, vol. 21, no. 6, pp. 1212–1219, 2022.

[S6] J.-Y. Li, Y.-H. Li, F. Zhang et al., "Visible-light-driven integrated organic synthesis and hydrogen evolution over 1D/2D CdS-$Ti_3C_2T_x$ MXene composites," Applied Catalysis B: Environmental, vol. 269, pp. 118783, 2020.

[S7] J. Wang, T. Xia, L. Wang et al., "Enabling visible-light-driven selective CO_2 reduction by doping quantum dots: trapping electrons and suppressing H_2 evolution," Angewandte Chemie International Edition, vol. 57, no. 50, pp. 16447–16451, 2018.

[S8] M. Yang, Y. Wang, Y. Ren et al., "Zn/Cd ratio-dependent synthetic conditions in ternary ZnCdS quantum dots," Journal of Alloys and Compounds, vol. 752, pp. 260–266, 2018.

[S9] L. Yuan, Y.-H. Li, M.-Y. Qi et al., "Defect-promoted visible light-driven C-C coupling reactions pairing with CO_2 reduction," Journal of Catalysis, vol. 390, pp. 244–250, 2020.
